# Supplementary material for: Trade-offs in diagnostic algorithm, population coverage, and duration of community screening for tuberculosis: A modelling study
Source: PLoS Med. 2026 Apr 6;23(4):e1005040. doi: 10.1371/journal.pmed.1005040 (PMC13068317; doi:10.1371/journal.pmed.1005040)
Supplement: S1 Appendix — Additional methodological details and results. (DOCX) [file pmed.1005040.s001.docx]

**Exploring trade-offs in diagnostic algorithm, population coverage, and duration of community screening for tuberculosis**

Katherine C. Horton, Alexandra S. Richards, Alvaro Schwalb, Rein M. G. J. Houben

**SUPPLEMENTAL MATERIALS**

1. **Model equations**

The base model without screening is described by the following set of equations:

$$N(t)=S(t)+I(t)+C(t)+Rn(t)+nTB(t)+aTB(t)+sTB(t)+Tp(t)+Rt(t)$$

$$\lambda(t)=\left( \beta*\frac{\left( t*aTB(t)+sTB(t) \right)}{N(t)} \right)$$

$$\omega(t)=\mu*N(t)+\mu_{sTB}*sTB(t)$$

$$\frac{dS(t)}{dt}=-\left( \lambda\left( t \right)+\mu\right)*S(t)+\omega(t)$$

$$\frac{dC(t)}{dt}=-\left( \lambda(t)+\mu\right)*C(t)+infclr*I(t)$$

$$\frac{dRn(t)}{dt}=-\left( \lambda(t)*p+\mu\right)*Rn(t)+ntbrec*nTB(t)$$

$$\frac{dI(t)}{dt}=-\left( infclr+infntb+infatb+\mu\right)*I(t)+\lambda(t)*(S(t)+p*Rn(t)+r*Rt(t))$$

$$\frac{dnTB(t)}{dt}=-\left( ntbrec+ntbatb+\mu\right)*nTB(t)+infntb*I(t)+atbntb*aTB(t)$$

$$\frac{daTB(t)}{dt}=-\left( atbntb+atbstb+\mu\right)*aTB(t)+infatb*I(t)+ntbatb*nTB(t)+stbatb*sTB(t)$$

$$\frac{dsTB(t)}{dt}=-\left( stbatb+stbtrt+\mu+\mu_{sTB} \right)*sTB(t)+atbstb*aTB(t)$$

$$\frac{dTp(t)}{dt}=-\left( trtrec+\mu\right)*Tp(t)+stbtrt*sTB(t)$$

$$\frac{dRt(t)}{dt}=-\left( \lambda(t)*r+\mu\right)*Rt(t)+trtrec*Tp(t)$$

1. **Model calibration**

Table A: Prior and posterior parameters for each calibration

| Parameter | Description | Prior median and range | Posterior median and range | | | Ref. |
| --- | --- | --- | --- | --- | --- | --- |
|  |  |  | Prevalence  1,000/100,000 | Prevalence  500/100,000 | Prevalence  250/100,000 |  |
| beta | Transmission coefficient | 6  (0-12) | 2.55  (1.79-3.19) | 2.83  (2.42-3.26) | 1.61  (1.37-1.86) | - |
| p | Relative risk of infection following recovery from non-infectious TB | 0.22 (0.14-0.30) | 0.20  (0.16-0.26) | 0.22  (0.17-0.27) | 0.20  (0.17-0.23) | [1] |
| r | Relative risk of infection for previously treated individuals | 3.21 (2.14-4.27) | 2.99  (2.26-3.93) | 3.24  (2.49-3.93) | 3.09  (2.30-4.11) | [2] |
| t | Transmission from asymptomatic infectious TB relative to symptomatic infectious TB | 0.81 (0.62-1.00) | 0.78  (0.65-0.93) | 0.87  (0.75-0.96) | 0.80  (0.71-0.89) | [3] |
| infclr | Rate of clearance from infection | 2.12 (0.93-3.30) | 2.11  (1.40-2.77) | 2.57  (2.02-3.11) | 1.95  (1.65-2.35) | [4] |
| infntb | Rate of progression from infection  to non-infectious TB | 0.14 (0.04-0.23) | 0.11  (0.06-0.16) | 0.09  (0.06-0.13) | 0.11  (0.08-0.13) | [4] |
| infatb | Rate of progression from infection  to asymptomatic infectious TB | 0.06 (0.01-0.10) | 0.04  (0.02-0.05) | 0.03  (0.02-0.05) | 0.06  (0.04-0.08) | [4] |
| ntbrec | Rate of recovery from  non-infectious TB | 0.19 (0.14-0.23) | 0.19  (0.16-0.21) | 0.18  (0.16-0.21) | 0.18  (0.16-0.20) | [4] |
| ntbatb | Rate of progression from  non-infectious TB  to asymptomatic infectious TB | 0.25 (0.21-0.28) | 0.25  (0.23-0.27) | 0.25  (0.22-0.27) | 0.24  (0.22-0.25) | [4] |
| atbntb | Rate of recovery from subclinical to non-infectious TB | 1.64 (1.24-2.03) | 1.64  (1.45-1.86) | 1.67  (1.45-1.89) | 1.71  (1.52-1.86) | [4] |
| atbstb | Rate of progression from asymptomatic infectious TB to symptomatic infectious TB | 0.75  (0.56-0.94) | 0.77  (0.65-0.90) | 0.75  (0.65-0.86) | 0.75  (0.64-0.87) | [4] |
| stbatb | Rate of recovery from symptomatic infectious TB to asymptomatic infectious TB | 0.59 (0.46-0.72) | 0.58  (0.49-0.68) | 0.60  (0.51-0.70) | 0.56  (0.47-0.64) | [4] |
| stbtrt | Rate of routine treatment initiation from symptomatic infectious TB | 0.67  (0.57-0.77) | 0.68  (0.60-0.74) | 0.68  (0.61-0.75) | 0.68  (0.62-0.73) | [5] |
| trtrec | Rate of treatment completion | 2.00 | 2.00 | 2.00 | 2.00 | [6] |
| μ_sTB_ | TB-specific mortality rate | 0.33 (0.28-0.38) | 0.33  (0.29-0.36) | 0.32  (0.30-0.35) | 0.32  (0.29-0.35) | [4] |
| μ | Background mortality rate | 0.0137 | 0.0137 | 0.0137 | 0.0137 | [7] |

Table B: Epidemiological estimates from models calibrated to infectious TB prevalence levels of 1,000, 500, and 250 per 100,000 population (± 10%). Table shows median values and 95% uncertainty intervals. infTB: infectious TB; sTB: symptomatic infectious TB; nTB: non-infectious TB.


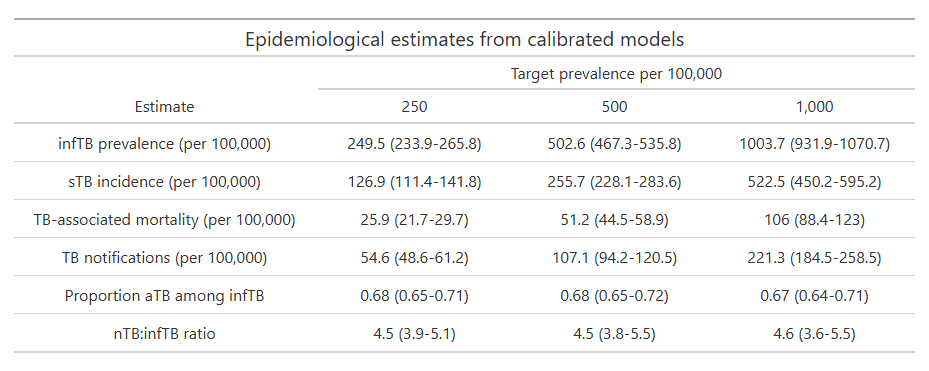


1. **Screening implementation**

Screening implementation required extending the base model (see Supplemental Materials Model Equations) to include four additional “on treatment via screening” compartments (see Figure 1), as well as a second parallel set of compartments and equations described in greater detail below.

For screening implementation, equations for the total population ($N(t)$), force of infection ($\lambda(t)$), and model entry ($\omega(t)$) were expanded to include relevant states from both sets of compartments.

$$N\left( t \right)=S\left( t \right)+I\left( t \right)+C\left( t \right)+Rn\left( t \right)+nTB\left( t \right)+aTB\left( t \right)+sTB\left( t \right)+Tp\left( t \right)+Ti\left( t \right)+Tc\left( t \right)+Tr\left( t \right)+Td\left( t \right)+Rt\left( t \right)+Sx\left( t \right)+Ix\left( t \right)+Cx\left( t \right)+Rnx\left( t \right)+nTBx\left( t \right)+aTBx\left( t \right)+sTBx\left( t \right)+Tpx\left( t \right)+Tix\left( t \right)+Tcx\left( t \right)+Trx\left( t \right)+Tdx\left( t \right)+Rtx\left( t \right)$$

$$\lambda\left( t \right)=\left( \beta*\frac{\left( t*aTB\left( t \right)+sTB\left( t \right)+t*aTBx\left( t \right)+sTBx\left( t \right) \right)}{N\left( t \right)} \right)$$

$$\omega(t)=\mu*N(t)+\mu_{sTB}*\left( sTB\left( t \right)+sTBx\left( t \right) \right)$$

The following set of equations describes the extended base model (here after referred to as “base compartments”). All individuals enter the model in this set of equations. During screening, these equations track individuals not yet considered for screening.

$$\frac{dS\left( t \right)}{dt}=-\left( \lambda\left( t \right)+\mu\right)*S\left( t \right)+\omega(t)+trtrec*Ti\left( t \right)$$

$$\frac{dC\left( t \right)}{dt}=-\left( \lambda\left( t \right)+\mu\right)*C\left( t \right)+infclr*I\left( t \right)+trtrec*Tc\left( t \right)$$

$$\frac{dRn\left( t \right)}{dt}=-\left( \lambda\left( t \right)*p+\mu\right)*Rn\left( t \right)+ntbrec*nTB\left( t \right)+trtrec*Tr\left( t \right)$$

$$\frac{dI\left( t \right)}{dt}=-\left( infclr+infntb+infatb+\mu\right)*I\left( t \right)+\lambda\left( t \right)*\left( S\left( t \right)+p*Rn\left( t \right)+r*Rt\left( t \right) \right)$$

$$\frac{dnTB\left( t \right)}{dt}=-\left( ntbrec+ntbatb+\mu\right)*nTB\left( t \right)+infntb*I\left( t \right)+atbntb*aTB\left( t \right)$$

$$\frac{daTB\left( t \right)}{dt}=-\left( atbntb+atbstb+\mu\right)*aTB\left( t \right)+infatb*I\left( t \right)+ntbatb*nTB\left( t \right)+stbatb*sTB\left( t \right)$$

$$\frac{dsTB\left( t \right)}{dt}=-\left( stbatb+stbtrt+\mu+\mu_{sTB} \right)*sTB\left( t \right)+atbstb*aTB\left( t \right)$$

$$\frac{dTp\left( t \right)}{dt}=-\left( trtrec+\mu\right)*Tp\left( t \right)+stbtrt*sTB\left( t \right)$$

$$\frac{dTi\left( t \right)}{dt}=-\left( trtrec+\mu\right)*Ti\left( t \right)$$

$$\frac{dTc\left( t \right)}{dt}=-\left( trtrec+\mu\right)*Tc\left( t \right)$$

$$\frac{dTr\left( t \right)}{dt}=-\left( trtrec+\mu\right)*Tr\left( t \right)$$

$$\frac{dTd\left( t \right)}{dt}=-\left( trtrec+\mu\right)*Td\left( t \right)$$

$$\frac{dRt\left( t \right)}{dt}=-\left( \lambda\left( t \right)*r+\mu\right)*Rt\left( t \right)+trtrec*Tp\left( t \right)$$

The following set of equations describes individuals who have been considered for screening (hereafter referred to as “screening compartments”). Compartments and equations are identical to the extended base model above, with “x” added to compartment names to distinguish the two sets of compartments and equations. During screening, these equations track individuals considered for screening.

$$\frac{dSx\left( t \right)}{dt}=-\lambda\left( t \right)*Sx\left( t \right)+trtrec*Tix(t)$$

$$\frac{dCx\left( t \right)}{dt}=-\left( \lambda\left( t \right)+\mu\right)*Cx\left( t \right)+infclr*Ix\left( t \right)+trtrec*Tcx\left( t \right)$$

$$\frac{dRnx\left( t \right)}{dt}=-\left( \lambda\left( t \right)*p+\mu\right)*Rnx\left( t \right)+ntbrec*nTBx\left( t \right)+trtrec*Trx\left( t \right)$$

$$\frac{dIx\left( t \right)}{dt}=-\left( infclr+infntb+infatb+\mu\right)*Ix\left( t \right)+\lambda\left( t \right)*\left( Sx\left( t \right)+p*Rnx\left( t \right)+r*Rtx\left( t \right) \right)$$

$$\frac{dnTBx\left( t \right)}{dt}=-\left( ntbrec+ntbatb+\mu\right)*nTBx\left( t \right)+infntb*Ix\left( t \right)+atbntb*aTBx\left( t \right)$$

$$\frac{daTBx\left( t \right)}{dt}=-\left( atbntb+atbstb+\mu\right)*aTBx\left( t \right)+infatb*Ix\left( t \right)+ntbatb*nTBx\left( t \right)+stbatb*sTBx\left( t \right)$$

$$\frac{dsTBx\left( t \right)}{dt}=-\left( stbatb+stbtrt+\mu+\mu_{sTB} \right)*sTBx\left( t \right)+atbstb*aTBx\left( t \right)$$

$$\frac{dTpx\left( t \right)}{dt}=-\left( trtrec+\mu\right)*Tpx\left( t \right)+stbtrt*sTBx\left( t \right)$$

$$\frac{dTix\left( t \right)}{dt}=-\left( trtrec+\mu\right)*Tix\left( t \right)$$

$$\frac{dTcx\left( t \right)}{dt}=-\left( trtrec+\mu\right)*Tcx\left( t \right)$$

$$\frac{dTrx\left( t \right)}{dt}=-\left( trtrec+\mu\right)*Trx\left( t \right)$$

$$\frac{dTdx\left( t \right)}{dt}=-\left( trtrec+\mu\right)*Tdx\left( t \right)$$

$$\frac{dRtx(t)}{dt}=-\left( \lambda(t)*r+\mu\right)*Rtx(t)+trtrec*Tpx(t)$$

Screening was implemented using the deSolve event function [8], which interrupts integration routines to allow changes in model states at designated timepoints. During each month of a 12-month round of screening, one-twelfth of each base compartment was considered eligible for screening and moved from base compartments to screening compartments using this function.

A proportion of these moved to one of four “on treatment via screening” compartments (Ts for Susceptible; Ti for Infection and Cleared; Tr for Recovered; Td for TB states and Previously treated), determined by population coverage and diagnostic algorithm. The diagnostic algorithm was defined using probability of a positive test for each tool. Probabilities of a positive test were broadly based on sensitivity for sTB and specificity for non-TB states [9-12], complemented by data from national TB prevalence surveys [13, 14], with assumptions where no data were available (see Supplementary Table 3 and Supplementary Table 4).

$${scr}_{x}=population coverage*{probabilty of a positive test}_{x}$$

$$where x indicates the base compartment from which individuals were screened$$

The remaining proportion moved from base compartment to screening compartments without changing state (e.g., S to Sx). This included individuals not included in population coverage and those who were screened but tested negative.

At the end of a round of screening, all individuals in screening compartments were moved to base compartments, without changing state (e.g., Sx to S).

Table C: Summary of probability of a positive test result for each tool used in diagnostic algorithms by model state

| Model state | Probability of a positive test result | | | |
| --- | --- | --- | --- | --- |
|  | Prolonged cough | Chest x-ray | Xpert Ultra (screening use) | Xpert Ultra (confirmatory use) |
| Susceptible | 0.047-0.074 | 0.069-0.134 | 0.005-0.008 | 0.026-0.070 |
| Infected | 0.047-0.074 | 0.069-0.134 | 0.005-0.008 | 0.026-0.070 |
| Cleared | 0.047-0.074 | 0.069-0.134 | 0.005-0.008 | 0.026-0.070 |
| Recovered | 0.089-0.162 | 0.481-0.524 | 0.020-0.060 | 0.034-0.295 |
| Non-infectious TB | 0.129-0.249 | 0.626-0.712 | 0.026-0.070 | 0.026-0.070 |
| Asymptomatic infectious TB | 0.000 | 0.626-0.712 | 0.676-0.856 | 0.676-0.856 |
| Symptomatic infectious TB | 0.900-1.000 | 0.770-0.900 | 0.862-0.947 | 0.862-0.947 |
| Treated | 0.089-0.162 | 0.481-0.524 | 0.020-0.060 | 0.034-0.295 |

Table D: Probability of a positive test result for each screening diagnostic tool for each state in the model:

| Model state | Probability of a positive test range | Description | Reference |
| --- | --- | --- | --- |
| Prolonged cough | | | |
| Susceptible | 0.047-0.074 | Interquartile range for the proportion of national TB prevalence survey participants reporting prolonged (≥ 2 weeks) cough, regardless of CXR or TB status | [9, 13] |
| Infected | 0.047-0.074 | Interquartile range for the proportion of national TB prevalence survey participants reporting prolonged (≥ 2 weeks) cough, regardless of CXR or TB status | [9, 13] |
| Cleared | 0.047-0.074 | Interquartile range for the proportion of national TB prevalence survey participants reporting prolonged (≥ 2 weeks) cough, regardless of CXR or TB status | [9, 13] |
| Recovered | 0.089-0.162 | Midpoint between bounds for Susceptible/ Infected/Cleared and Non-infectious TB | Assumption |
| Non-infectious TB | 0.129-0.249 | Interquartile range for the proportion of national TB prevalence survey participants reporting prolonged (≥ 2 weeks) cough with abnormal CXR, regardless of TB status | [13] |
| Asymptomatic infectious TB | 0.00 | Assumption based on state definition | Assumption |
| Symptomatic infectious TB | 0.900-1.000 | Assumption based on state definition, with uncertainty recognising that individuals may report a symptom other than prolonged cough | Assumption |
| Treated | 0.089-0.162 | Midpoint between bounds for Susceptible/ Infected/Cleared and Non-infectious TB | Assumption |
| Chest X-ray | | | |
| Susceptible | 0.069-0.134 | Interquartile range for the proportion of national TB prevalence survey participants with abnormal CXR, regardless of TB status | [13] |
| Infected | 0.069-0.134 | Interquartile range for the proportion of national TB prevalence survey participants with abnormal CXR, regardless of TB status | [13] |
| Cleared | 0.069-0.134 | Interquartile range for the proportion of national TB prevalence survey participants with abnormal CXR, regardless of TB status | [13] |
| Recovered | 0.481-0.524 | Interquartile range for proportion with abnormal CXR suggestive of TB among participants of national TB prevalence survey reporting TB history | [14] |
| Non-infectious TB | 0.626-0.712 | Midpoint between the bounds for Recovered/Treated and Symptomatic infectious TB | Assumption |
| Asymptomatic infectious TB | 0.626-0.712 | Midpoint between the bounds for Recovered/Treated and Symptomatic infectious TB | Assumption |
| Symptomatic infectious TB | 0.770-0.900 | Sensitivity of CXR suggestive for TB for bacteriologically confirmed TB in screening use case | [9] |
| Treated | 0.481-0.524 | Interquartile range for proportion with abnormal CXR suggestive of TB among participants of national TB prevalence survey reporting TB history | [14] |
| Xpert Ultra: Screening use | | | |
| Susceptible | 0.005-0.008 | (1 – specificity) for individuals in a community in Kampala, Uganda | [12] |
| Infected | 0.005-0.008 | (1 – specificity) for individuals in a community in Kampala, Uganda | [12] |
| Cleared | 0.005-0.008 | (1 – specificity) for individuals in a community in Kampala, Uganda | [12] |
| Recovered | 0.020-0.060 | (1 – specificity) for individuals screened positive for symptoms and/or CXR with a history of TB in the community | [10] |
| Non-infectious TB | 0.026-0.070 | (1 – specificity) for pulmonary TB from individuals in primary care facilities and local hospitals | [11] |
| Asymptomatic infectious TB | 0.676-0.856 | Sensitivity for smear-negative TB from individuals in primary care facilities and local hospitals | [11] |
| Symptomatic infectious TB | 0.862-0.947 | Sensitivity for pulmonary TB from individuals in primary care facilities and local hospitals | [11] |
| Treated | 0.020-0.060 | (1 – specificity) for individuals screened positive for symptoms and/or CXR with a history of TB in the community | [10] |
| Xpert Ultra: Confirmatory use | | | |
| Susceptible | 0.026-0.070 | (1 – specificity) for pulmonary TB from individuals in primary care facilities and local hospitals | [11] |
| Infected | 0.026-0.070 | (1 – specificity) for pulmonary TB from individuals in primary care facilities and local hospitals | [11] |
| Cleared | 0.026-0.070 | (1 – specificity) for pulmonary TB from individuals in primary care facilities and local hospitals | [11] |
| Recovered | 0.034-0.295 | (1 – specificity) in individuals with history of TB treatment in primary care facilities and local hospitals | [11] |
| Non-infectious TB | 0.026-0.070 | (1 – specificity) for pulmonary TB from individuals in primary care facilities and local hospitals | [11] |
| Asymptomatic infectious TB | 0.676-0.856 | Sensitivity for smear-negative TB from individuals in primary care facilities and local hospitals | [11] |
| Symptomatic infectious TB | 0.862-0.947 | Sensitivity for pulmonary TB from individuals in primary care facilities and local hospitals | [11] |
| Treated | 0.034-0.295 | (1 – specificity) in individuals with history of TB treatment in primary care facilities and local hospitals | [11] |

**III. Results**


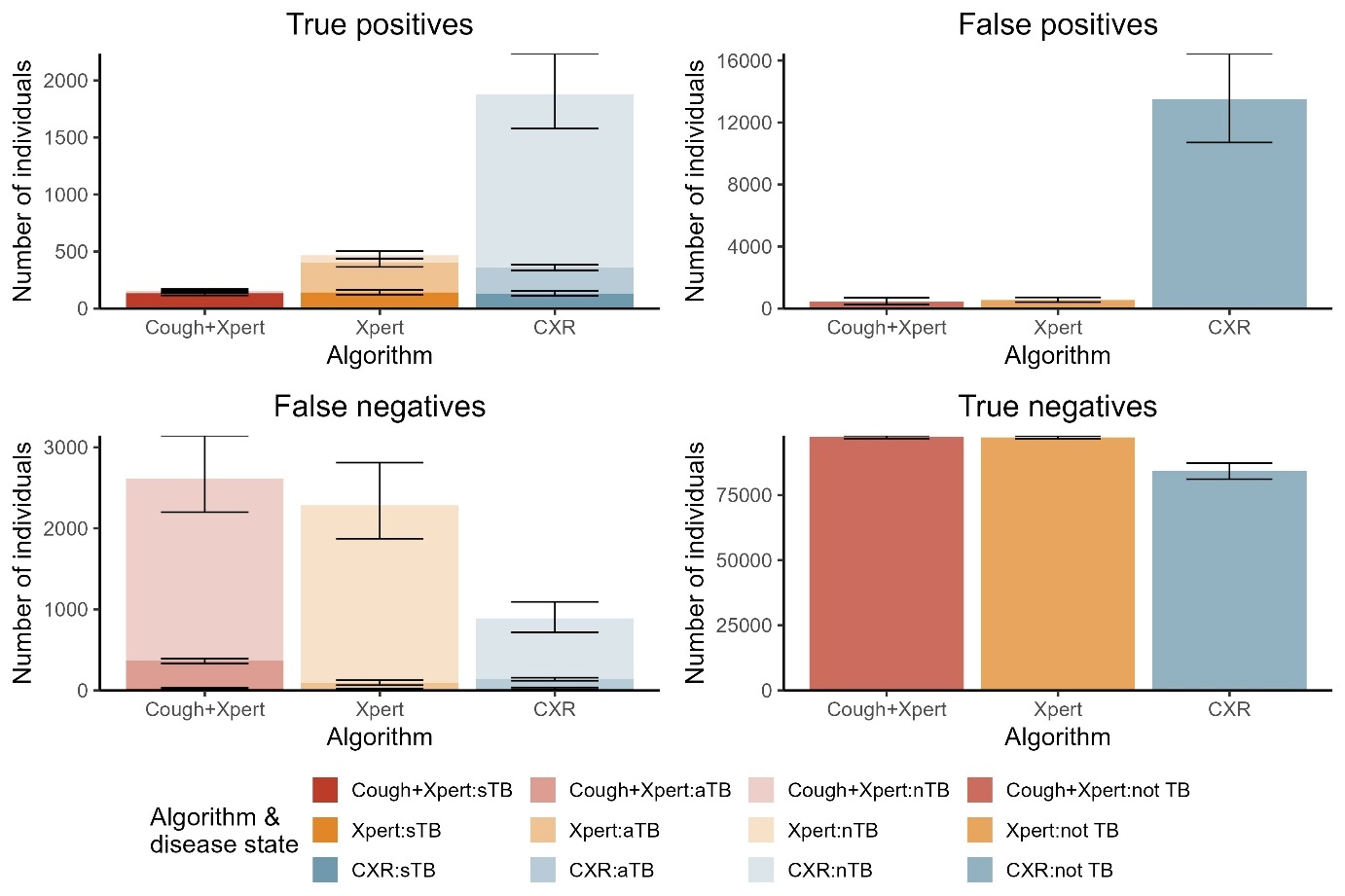


Fig A: Matrix showing number of individuals with true positive, false positive, false negative, and true negative results by diagnostic algorithm for one round of screening with 100% coverage in a population with baseline prevalence of 500 per 100,000. TB states are disaggregated to show symptomatic infectious TB (sTB), asymptomatic infectious TB (aTB), and non-infectious TB (nTB). Note y-axis differs in each quadrant.

Table E: Number of individuals who tested positive for TB in one round of screening with 100% coverage in a population with baseline prevalence of 500 per 100,000. sTB: symptomatic infectious TB; aTB: asymptomatic infectious TB; nTB: non-infectious TB; infectious TB: sTB+aTB; all TB: sTB+aTB+nTB.


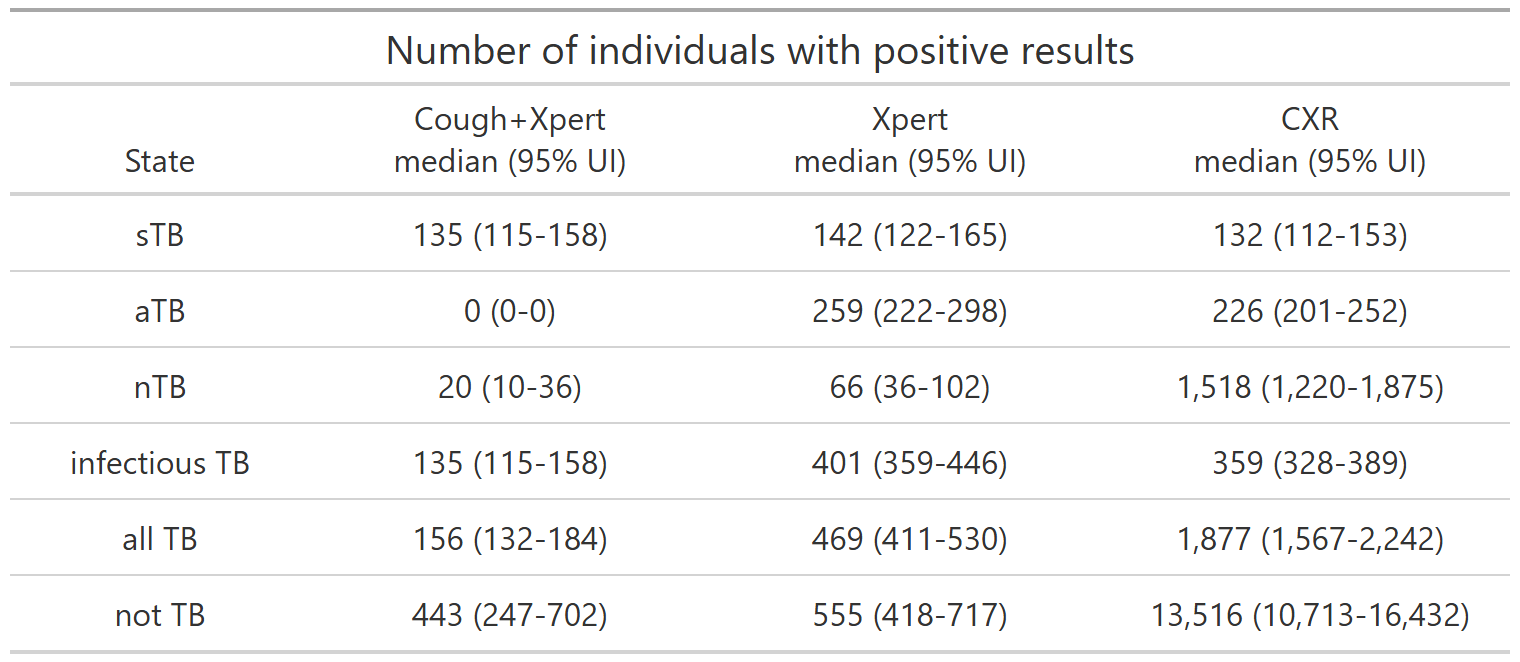


Table F: Number of individuals who tested negative for TB in one round of screening with 100% coverage in a population with baseline prevalence of 500 per 100,000. sTB: symptomatic infectious TB; aTB: asymptomatic infectious TB; nTB: non-infectious TB; infectious TB: sTB+aTB; all TB: sTB+aTB+nTB.


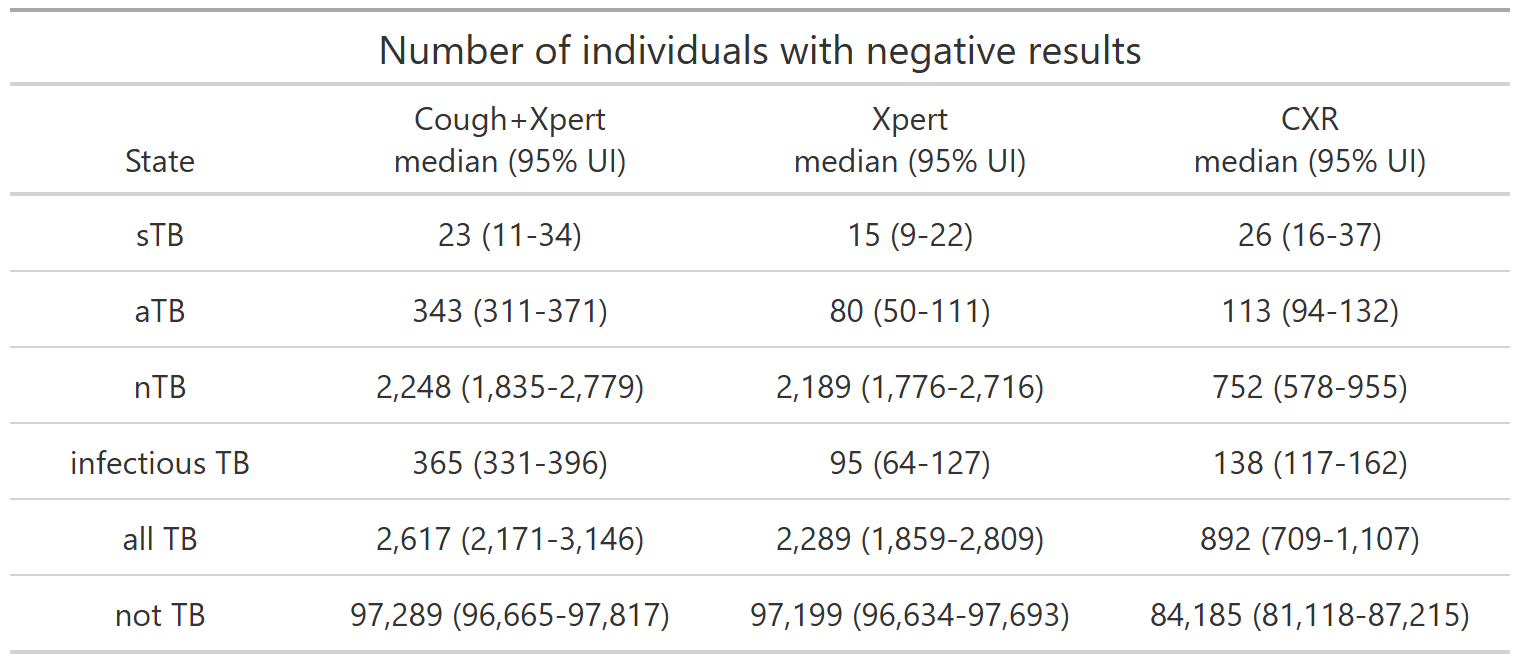


Table G: Number of individuals with true positive results in each round of screening by diagnostic algorithm and population coverage for a population with baseline prevalence of 500 per 100,000.

|  | Number of individuals with true positive results | | | | | | | | | | |
| --- | --- | --- | --- | --- | --- | --- | --- | --- | --- | --- | --- |
|  | Population coverage | | | | | | | | | | |
| Round | | 10% | 20% | 30% | 40% | 50% | 60% | 70% | 80% | 90% | 100% |
| Algorithm targeting symptomatic infectious TB (Cough+Xpert) | | | | | | | | | | | |
| 1 | | 16  (13-19) | 31  (27-37) | 47  (40-55) | 63  (53-74) | 78  (66-93) | 93  (79-111) | 109  (91-128) | 125  (106-150) | 141  (118-169) | 156  (133-187) |
| 2 | | 15  (13-18) | 30  (25-35) | 44  (37-51) | 57  (48-67) | 69  (58-82) | 81  (69-95) | 92  (78-107) | 102  (88-122) | 112  (95-133) | 121  (103-142) |
| 3 | | 15  (13-18) | 29  (25-34) | 42  (36-50) | 55  (46-64) | 66  (56-78) | 77  (65-90) | 87  (74-101) | 96  (83-114) | 105  (89-124) | 112  (96-132) |
| 4 | | 15  (13-18) | 29  (25-34) | 41  (36-49) | 53  (45-63) | 64  (54-75) | 74  (63-86) | 83  (71-96) | 91  (79-108) | 99  (85-117) | 106  (91-124) |
| 5 | | 15  (13-18) | 29  (24-33) | 41  (35-48) | 52  (44-61) | 62  (53-73) | 71  (61-83) | 79  (68-92) | 87  (75-103) | 94  (81-110) | 100  (87-116) |
| Algorithm targeting infectious TB (Xpert) | | | | | | | | | | | |
| 1 | | 47  (41-54) | 95  (82-107) | 142  (124-160) | 188  (165-213) | 236  (205-267) | 282  (247-318) | 326  (290-375) | 375  (331-426) | 421  (365-475) | 468  (405-527) |
| 2 | | 46  (40-52) | 88  (77-100) | 128  (112-144) | 163  (143-185) | 196  (170-223) | 225  (198-255) | 250  (220-288) | 274  (240-315) | 294  (255-339) | 311  (269-357) |
| 3 | | 44  (39-51) | 84  (73-95) | 119  (104-134) | 148  (129-168) | 174  (152-200) | 195  (172-223) | 213  (186-245) | 228  (199-264) | 241  (208-282) | 250  (215-293) |
| 4 | | 43  (38-50) | 80  (70-91) | 111  (97-126) | 136  (119-155) | 156  (136-180) | 172  (151-198) | 185  (161-214) | 194  (167-226) | 201  (171-238) | 206  (175-243) |
| 5 | | 43  (38-49) | 77  (67-88) | 104  (92-118) | 125  (109-143) | 141  (122-164) | 152  (133-177) | 161  (139-187) | 165  (141-195) | 168  (142-202) | 168  (142-203) |
| Algorithm targeting all TB (CXR) | | | | | | | | | | | |
| 1 | | 188  (156-226) | 378  (312-453) | 567  (467-680) | 753  (619-914) | 937  (782-1132) | 1124  (933-1367) | 1309  (1099-1601) | 1497  (1233-1820) | 1688  (1410-2032) | 1872  (1556-2255) |
| 2 | | 176  (146-212) | 329  (274-395) | 458  (380-553) | 562  (465-681) | 640  (536-768) | 696  (586-837) | 728  (611-879) | 738  (617-885) | 723  (608-869) | 687  (574-828) |
| 3 | | 165  (137-198) | 289  (240-346) | 371  (309-448) | 421  (351-508) | 441  (368-530) | 436  (366-523) | 411  (343-494) | 370  (309-448) | 319  (261-394) | 262  (212-327) |
| 4 | | 155  (129-186) | 253  (211-304) | 303  (253-364) | 317  (263-381) | 305  (256-369) | 275  (230-329) | 234  (192-284) | 188  (153-233) | 142  (113-182) | 101  (77-133) |
| 5 | | 146  (121-175) | 222  (185-268) | 248  (207-298) | 240  (200-289) | 212  (177-257) | 175  (144-211) | 133  (108-166) | 96  (76-122) | 63  (48-86) | 39  (28-55) |

Table H: Number of individuals with false positive results in each round of screening by diagnostic algorithm and population coverage for a population with baseline prevalence of 500 per 100,000.

|  | Number of individuals with false positive results | | | | | | | | | |
| --- | --- | --- | --- | --- | --- | --- | --- | --- | --- | --- |
|  | Population coverage | | | | | | | | | |
| Round | 10% | 20% | 30% | 40% | 50% | 60% | 70% | 80% | 90% | 100% |
| Algorithm targeting symptomatic infectious TB (Cough+Xpert) | | | | | | | | | | |
| 1 | 44  (23-67) | 87  (48-135) | 132  (70-202) | 174  (93-277) | 225  (116-349) | 265  (138-414) | 306  (168-468) | 353  (189-559) | 391  (221-611) | 436  (235-685) |
| 2 | 44  (23-67) | 87  (48-135) | 132  (70-202) | 174  (93-277) | 226  (116-350) | 266  (139-415) | 307  (169-469) | 354  (190-560) | 392  (222-613) | 439  (237-688) |
| 3 | 44  (23-67) | 87  (48-135) | 132  (70-202) | 175  (93-278) | 226  (116-351) | 266  (139-416) | 308  (169-470) | 356  (191-562) | 394  (223-616) | 441  (239-690) |
| 4 | 44  (23-67) | 87  (48-135) | 132  (70-202) | 175  (93-278) | 227  (116-352) | 267  (139-417) | 308  (170-471) | 357  (191-563) | 395  (224-619) | 443  (240-692) |
| 5 | 44  (23-67) | 87  (48-135) | 132  (70-203) | 175  (93-278) | 227  (116-353) | 268  (140-418) | 309  (170-471) | 358  (191-564) | 396  (224-620) | 444  (241-694) |
| Algorithm targeting infectious TB (Xpert) | | | | | | | | | | |
| 1 | 57  (42-72) | 112  (85-143) | 169  (125-214) | 223  (165-289) | 280  (212-364) | 334  (249-441) | 393  (300-503) | 448  (342-570) | 500  (378-653) | 559  (421-716) |
| 2 | 57  (42-72) | 112  (85-144) | 169  (126-215) | 224  (167-290) | 281  (214-367) | 337  (252-445) | 398  (302-509) | 454  (346-577) | 509  (385-661) | 569  (428-725) |
| 3 | 57  (42-72) | 113  (85-144) | 170  (126-216) | 226  (168-291) | 284  (216-369) | 340  (254-448) | 401  (305-513) | 459  (348-583) | 515  (391-669) | 575  (436-732) |
| 4 | 57  (42-73) | 113  (86-144) | 171  (127-217) | 227  (169-292) | 285  (217-370) | 342  (255-450) | 404  (308-516) | 462  (350-587) | 519  (394-674) | 580  (439-738) |
| 5 | 57  (42-73) | 113  (86-145) | 171  (127-217) | 227  (169-293) | 286  (218-372) | 343  (256-452) | 405  (309-517) | 464  (351-590) | 522  (396-677) | 583  (441-743) |
| Algorithm targeting all TB (CXR) | | | | | | | | | | |
| 1 | 1372  (1070-1645) | 2714  (2135-3269) | 4053  (3223-4912) | 5436  (4313-6518) | 6842  (5406-8208) | 8049  (6431-9822) | 9497  (7496-11457) | 10911  (8536-13057) | 12316  (9734-14777) | 13592  (10620-16510) |
| 2 | 1372  (1071-1646) | 2716  (2142-3270) | 4056  (3221-4911) | 5437  (4320-6515) | 6859  (5421-8208) | 8090  (6473-9841) | 9516  (7555-11478) | 10958  (8621-13078) | 12393  (9832-14816) | 13676  (10766-16555) |
| 3 | 1376  (1074-1651) | 2735  (2155-3290) | 4091  (3256-4953) | 5502  (4369-6586) | 6946  (5499-8301) | 8205  (6576-9993) | 9668  (7702-11664) | 11135  (8768-13308) | 12613  (10022-15067) | 13899  (10943-16816) |
| 4 | 1379  (1077-1655) | 2746  (2165-3306) | 4117  (3275-4985) | 5547  (4393-6635) | 7004  (5545-8363) | 8276  (6643-10085) | 9739  (7771-11762) | 11222  (8847-13428) | 12695  (10077-15172) | 13975  (10992-16910) |
| 5 | 1380  (1078-1658) | 2754  (2173-3319) | 4132  (3280-5005) | 5571  (4405-6665) | 7031  (5564-8396) | 8307  (6677-10123) | 9771  (7796-11805) | 11247  (8859-13457) | 12702  (10075-15176) | 13976  (10979-16903) |


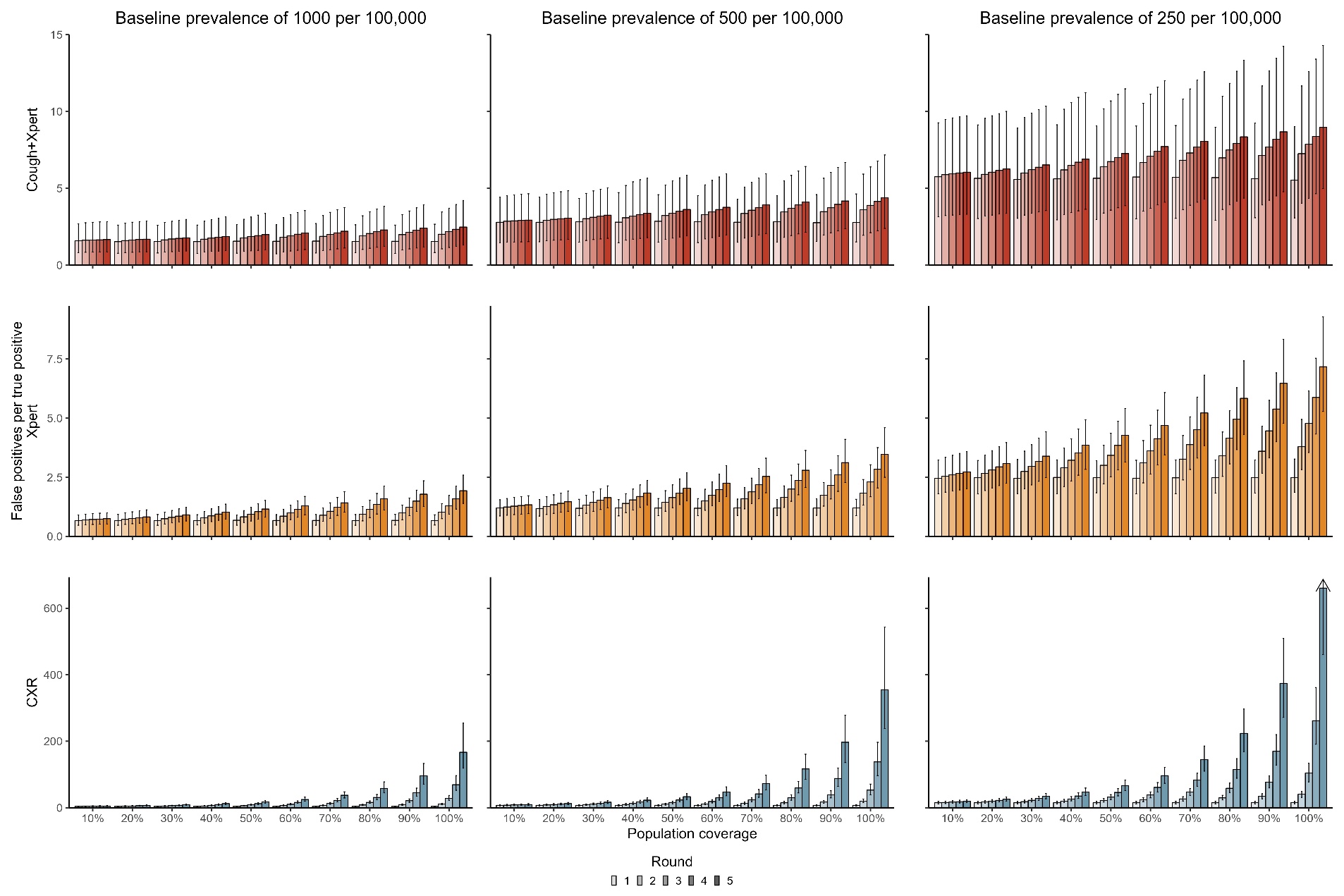


Fig B: Number of individuals with false positive results per individual with true positive results for each round of screening by diagnostic algorithm, population coverage, and baseline prevalence.

Table I: Number of individuals with false positive results per individual with true positive results for each round of screening by diagnostic algorithm and population coverage for a population with baseline prevalence of 500 per 100,000.

|  | Number of individuals with false positive results per individual with true positive results | | | | | | | | | |
| --- | --- | --- | --- | --- | --- | --- | --- | --- | --- | --- |
|  | Population coverage | | | | | | | | | |
| Round | 10% | 20% | 30% | 40% | 50% | 60% | 70% | 80% | 90% | 100% |
| Algorithm targeting symptomatic infectious TB (Cough+Xpert) | | | | | | | | | | |
| 1 | 2.8  (1.4-4.4) | 2.8  (1.5-4.4) | 2.8  (1.5-4.3) | 2.8  (1.5-4.7) | 2.8  (1.5-4.6) | 2.8  (1.5-4.5) | 2.8  (1.5-4.3) | 2.8  (1.5-4.5) | 2.8  (1.5-4.6) | 2.8  (1.5-4.6) |
| 2 | 2.9  (1.5-4.5) | 2.9  (1.6-4.6) | 3.0  (1.6-4.7) | 3.1  (1.6-5.2) | 3.2  (1.7-5.2) | 3.3  (1.7-5.2) | 3.4  (1.8-5.1) | 3.5  (1.8-5.5) | 3.5  (2.0-5.6) | 3.6  (1.9-5.9) |
| 3 | 2.9  (1.5-4.6) | 3.0  (1.6-4.7) | 3.1  (1.7-4.8) | 3.2  (1.7-5.4) | 3.4  (1.8-5.5) | 3.5  (1.8-5.5) | 3.6  (1.9-5.4) | 3.7  (1.9-5.8) | 3.7  (2.1-6.0) | 3.9  (2.1-6.4) |
| 4 | 2.9  (1.5-4.6) | 3.0  (1.6-4.8) | 3.2  (1.7-4.9) | 3.3  (1.7-5.6) | 3.5  (1.8-5.7) | 3.6  (1.9-5.7) | 3.7  (2.0-5.7) | 3.9  (2.0-6.1) | 4.0 (2.2-6.3) | 4.1  (2.2-6.8) |
| 5 | 2.9  (1.5-4.6) | 3.1  (1.7-4.8) | 3.2  (1.7-5.0) | 3.4  (1.8-5.7) | 3.6  (1.9-5.8) | 3.8  (2.0-5.9) | 3.9  (2.1-5.9) | 4.1  (2.1-6.4) | 4.2  (2.4-6.7) | 4.4  (2.4-7.2) |
| Algorithm targeting infectious TB (Xpert) | | | | | | | | | | |
| 1 | 1.2  (0.9-1.5) | 1.2  (0.9-1.6) | 1.2  (0.9-1.6) | 1.2  (0.9-1.6) | 1.2  (0.9-1.6) | 1.2  (0.9-1.6) | 1.2  (0.9-1.6) | 1.2  (0.9-1.6) | 1.2  (0.9-1.6) | 1.2  (0.9-1.6) |
| 2 | 1.2  (0.9-1.6) | 1.3  (0.9-1.7) | 1.3  (1.0-1.7) | 1.4  (1.0-1.8) | 1.4  (1.1-1.9) | 1.5  (1.1-2.0) | 1.6  (1.2-2.1) | 1.7  (1.2-2.1) | 1.7  (1.3-2.3) | 1.8  (1.3-2.4) |
| 3 | 1.3  (0.9-1.6) | 1.3  (1.0-1.8) | 1.4  (1.1-1.9) | 1.5  (1.1-2.0) | 1.6  (1.2-2.2) | 1.7  (1.3-2.3) | 1.9  (1.4-2.5) | 2.0  (1.5-2.6) | 2.2  (1.6-2.8) | 2.3  (1.7-3.0) |
| 4 | 1.3  (0.9-1.7) | 1.4  (1.0-1.8) | 1.5  (1.1-2.0) | 1.7  (1.2-2.2) | 1.8  (1.3-2.4) | 2.0  (1.5-2.6) | 2.2  (1.6-2.9) | 2.4  (1.8-3.1) | 2.6  (1.9-3.4) | 2.8  (2.0-3.8) |
| 5 | 1.3  (1.0-1.7) | 1.5  (1.1-1.9) | 1.6  (1.2-2.1) | 1.8  (1.3-2.4) | 2.0  (1.5-2.7) | 2.3  (1.7-3.0) | 2.5  (1.8-3.3) | 2.8  (2.1-3.6) | 3.1  (2.3-4.1) | 3.5  (2.5-4.6) |
| Algorithm targeting all TB (CXR) | | | | | | | | | | |
| 1 | 7.2  (5.4-9.4) | 7.2  (5.4-9.3) | 7.1  (5.4-9.3) | 7.1  (5.4-9.4) | 7.3  (5.4-9.3) | 7.1  (5.4-9.4) | 7.2  (5.4-9.4) | 7.2  (5.4-9.4) | 7.2  (5.5-9.3) | 7.2  (5.5-9.4) |
| 2 | 7.7  (5.7-10.1) | 8.2  (6.3-10.6) | 8.8  (6.7-11.4) | 9.6  (7.4-12.5) | 10.7  (8.0-13.5) | 11.5  (8.8-15.1) | 13.0  (9.9-16.8) | 14.7  (11.2-19.0) | 16.9  (12.9-21.8) | 19.7  (15.2-25.5) |
| 3 | 8.3  (6.1-10.7) | 9.5  (7.2-12.2) | 10.9  (8.3-14.1) | 12.8  (9.9-16.8) | 15.8  (11.7-20.0) | 18.7  (14.3-24.3) | 23.4  (17.9-30.5) | 29.7  (22.7-38.9) | 38.9  (29.5-50.9) | 52.7  (38.8-71.1) |
| 4 | 8.8  (6.6-11.4) | 10.8  (8.3-14.0) | 13.5  (10.3-17.4) | 17.1  (13.3-22.5) | 23  (17.3-29.0) | 29.9  (22.9-39.1) | 41.6  (31.3-54.7) | 59.2  (44.4-78.7) | 88.1  (63.9-119.4) | 137.7  (96.7-196.7) |
| 5 | 9.4  (7.0-12.2) | 12.3  (9.4-15.9) | 16.5  (12.6-21.3) | 22.7  (17.6-30.0) | 33.1  (24.8-42.3) | 47.3  (35.9-62.7) | 72.9  (53.6-97.7) | 116.5  (84.9-160.8) | 196.9  (135.6-278.5) | 354.9  (237.7-543.3) |

Table J: Number of individuals with false positive results per individual with true positive results for each round of screening by diagnostic algorithm and population coverage for a population with baseline prevalence of 1,000 per 100,000.

|  | Number of individuals with false positive results per individual with true positive results | | | | | | | | | |
| --- | --- | --- | --- | --- | --- | --- | --- | --- | --- | --- |
|  | Population coverage | | | | | | | | | |
| Round | 10% | 20% | 30% | 40% | 50% | 60% | 70% | 80% | 90% | 100% |
| Algorithm targeting symptomatic infectious TB (Cough+Xpert) | | | | | | | | | | |
| 1 | 1.6  (0.8-2.7) | 1.5  (0.8-2.6) | 1.5  (0.8-2.6) | 1.5  (0.8-2.6) | 1.6  (0.8-2.6) | 1.6  (0.7-2.6) | 1.6  (0.8-2.7) | 1.5  (0.8-2.6) | 1.5  (0.8-2.6) | 1.5  (0.8-2.7) |
| 2 | 1.6  (0.8-2.8) | 1.6  (0.8-2.7) | 1.6  (0.8-2.8) | 1.7  (0.8-2.9) | 1.8  (0.9-3.0) | 1.8  (0.9-3.1) | 1.9  (0.9-3.2) | 1.9  (1.0-3.2) | 2.0  (1.0-3.3) | 2.0  (1.1-3.4) |
| 3 | 1.6  (0.8-2.8) | 1.6  (0.8-2.8) | 1.7  (0.9-2.8) | 1.8  (0.9-3.0) | 1.9  (0.9-3.1) | 1.9  (0.9-3.3) | 2.0  (1.0-3.4) | 2.1  (1.1-3.5) | 2.1  (1.1-3.5) | 2.2  (1.1-3.7) |
| 4 | 1.6  (0.8-2.8) | 1.7  (0.8-2.8) | 1.7  (0.9-2.9) | 1.8  (0.9-3.1) | 1.9  (1.0-3.2) | 2.0  (1.0-3.4) | 2.1  (1.1-3.6) | 2.2  (1.2-3.6) | 2.3  (1.1-3.7) | 2.3  (1.2-3.9) |
| 5 | 1.7  (0.9-2.8) | 1.7  (0.9-2.9) | 1.8  (0.9-3.0) | 1.9  (0.9-3.1) | 2.0  (1.0-3.4) | 2.1  (1.0-3.5) | 2.2  (1.1-3.7) | 2.3  (1.2-3.8) | 2.4  (1.2-3.9) | 2.5  (1.3-4.2) |
| Algorithm targeting infectious TB (Xpert) | | | | | | | | | | |
| 1 | 0.7  (0.5-0.9) | 0.7  (0.5-0.9) | 0.7  (0.5-0.9) | 0.7  (0.5-0.9) | 0.7  (0.5-0.9) | 0.7  (0.5-0.9) | 0.7  (0.5-0.9) | 0.7  (0.5-0.9) | 0.7  (0.5-0.9) | 0.7  (0.5-0.9) |
| 2 | 0.7  (0.5-0.9) | 0.7  (0.5-1.0) | 0.7  (0.5-1.0) | 0.8  (0.6-1.1) | 0.8  (0.6-1.1) | 0.9  (0.6-1.1) | 0.9  (0.6-1.2) | 0.9  (0.7-1.3) | 1.0  (0.7-1.3) | 1.0  (0.7-1.4) |
| 3 | 0.7  (0.5-1.0) | 0.8  (0.5-1.0) | 0.8  (0.6-1.1) | 0.9  (0.6-1.2) | 0.9  (0.7-1.2) | 1  (0.7-1.3) | 1.1  (0.8-1.4) | 1.1  (0.8-1.5) | 1.2  (0.9-1.6) | 1.3  (0.9-1.7) |
| 4 | 0.7  (0.5-1.0) | 0.8  (0.6-1.1) | 0.9  (0.6-1.2) | 0.9  (0.7-1.3) | 1.0  (0.7-1.4) | 1.1  (0.8-1.5) | 1.2  (0.9-1.6) | 1.4  (0.9-1.8) | 1.5  (1.1-2.0) | 1.6  (1.2-2.1) |
| 5 | 0.7  (0.5-1.0) | 0.8  (0.6-1.1) | 0.9  (0.7-1.2) | 1.0  (0.7-1.4) | 1.2  (0.8-1.5) | 1.3  (0.9-1.7) | 1.4  (1.0-1.9) | 1.6  (1.1-2.1) | 1.8  (1.3-2.4) | 1.9  (1.4-2.6) |
| Algorithm targeting all TB (CXR) | | | | | | | | | | |
| 1 | 3.9  (3.1-5.0) | 3.9  (3.1-5.0) | 4.0  (3.0-5.0) | 3.9  (3.1-5.0) | 3.9  (3.1-4.9) | 3.9  (3.1-5.0) | 4.0  (3.1-5.0) | 3.9  (3.1-5.0) | 3.9  (3.0-5.0) | 4.0  (3.1-5.0) |
| 2 | 4.2  (3.3-5.3) | 4.5  (3.5-5.8) | 4.9  (3.8-6.2) | 5.3  (4.2-6.7) | 5.8  (4.6-7.2) | 6.4  (5.1-8.1) | 7.2  (5.7-9.0) | 8.1  (6.5-10.1) | 9.3  (7.4-11.7) | 11.0  (8.8-13.8) |
| 3 | 4.5  (3.5-5.7) | 5.2  (4.0-6.6) | 6.1  (4.7-7.6) | 7.2  (5.6-8.9) | 8.5  (6.8-10.6) | 10.2  (8.3-12.9) | 12.8  (10.2-15.9) | 16.1  (13.0-20.4) | 21.0  (16.5-26.5) | 28.3  (22.4-37.1) |
| 4 | 4.8  (3.7-6.0) | 5.9  (4.6-7.5) | 7.4  (5.8-9.3) | 9.5  (7.4-11.7) | 12.2  (9.8-15.1) | 16  (13.0-20.4) | 22.1  (17.6-27.5) | 31.0  (24.5-39.7) | 45.2  (34.9-58.8) | 69.3  (52.4-96.7) |
| 5 | 5.1  (4.0-6.4) | 6.6  (5.2-8.4) | 8.9  (7.0-11.2) | 12.3  (9.7-15.2) | 17.3  (13.8-21.5) | 24.7  (19.9-31.6) | 37.5  (29.2-47.8) | 58.4  (44.8-77.7) | 96.1  (70.7-132.9) | 166.9  (118.8-254.4) |

Table K: Number of individuals with false positive results per individual with true positive results for each round of screening by diagnostic algorithm and population coverage for a population with infectious prevalence of 250 per 100,000.

|  | Number of individuals with false positive results per individual with true positive results | | | | | | | | | |
| --- | --- | --- | --- | --- | --- | --- | --- | --- | --- | --- |
|  | Population coverage | | | | | | | | | |
| Round | 10% | 20% | 30% | 40% | 50% | 60% | 70% | 80% | 90% | 100% |
| Algorithm targeting symptomatic infectious TB (Cough+Xpert) | | | | | | | | | | |
| 1 | 5.7  (3.1-9.3) | 5.6  (3.0-9.1) | 5.6  (3.0-8.9) | 5.6  (2.9-9.1) | 5.6  (3.0-9.1) | 5.7  (3.0-9.0) | 5.7  (3.0-9.1) | 5.7  (2.9-9.0) | 5.6  (3.1-9.2) | 5.5  (3.1-9.0) |
| 2 | 5.9  (3.2-9.5) | 5.9  (3.2-9.5) | 6.0  (3.3-9.6) | 6.2  (3.3-10.1) | 6.4  (3.4-10.2) | 6.7  (3.5-10.5) | 6.8  (3.6-10.8) | 7.0  (3.6-11.0) | 7.1  (3.9-11.7) | 7.2  (4.0-11.7) |
| 3 | 5.9  (3.3-9.6) | 6.0  (3.3-9.7) | 6.2  (3.4-9.9) | 6.5  (3.4-10.6) | 6.7  (3.6-10.7) | 7.1  (3.7-11.1) | 7.3  (3.9-11.5) | 7.5  (3.9-11.8) | 7.7  (4.2-12.6) | 7.9  (4.3-12.6) |
| 4 | 6.0  (3.3-9.7) | 6.2  (3.3-9.9) | 6.4  (3.5-10.1) | 6.7  (3.5-10.9) | 7.0  (3.7-11.1) | 7.4  (3.9-11.6) | 7.7  (4.1-12.0) | 7.9  (4.1-12.6) | 8.2  (4.5-13.5) | 8.4  (4.6-13.4) |
| 5 | 6.0  (3.3-9.7) | 6.2  (3.4-10.0) | 6.5  (3.5-10.3) | 6.9  (3.6-11.2) | 7.3  (3.9-11.5) | 7.7  (4.1-12.0) | 8.1  (4.3-12.6) | 8.3  (4.4-13.3) | 8.7  (4.8-14.2) | 9.0  (5.0-14.3) |
| Algorithm targeting infectious TB (Xpert) | | | | | | | | | | |
| 1 | 2.5  (1.8-3.2) | 2.5  (1.8-3.2) | 2.5  (1.8-3.2) | 2.5  (1.8-3.2) | 2.5  (1.8-3.2) | 2.5  (1.8-3.2) | 2.5  (1.8-3.2) | 2.5  (1.8-3.2) | 2.5  (1.8-3.2) | 2.5  (1.9-3.3) |
| 2 | 2.5  (1.9-3.3) | 2.7  (1.9-3.4) | 2.7  (2.0-3.6) | 2.9  (2.1-3.7) | 3.0  (2.2-3.8) | 3.1  (2.3-4.1) | 3.3  (2.4-4.3) | 3.4  (2.5-4.4) | 3.6  (2.7-4.7) | 3.8  (2.8-4.9) |
| 3 | 2.6  (1.9-3.4) | 2.8  (2.0-3.6) | 3.0  (2.2-3.9) | 3.2  (2.3-4.1) | 3.4  (2.5-4.4) | 3.6  (2.6-4.7) | 3.9  (2.8-5.1) | 4.2  (3.1-5.3) | 4.5  (3.3-5.8) | 4.8  (3.5-6.2) |
| 4 | 2.7  (2.0-3.5) | 2.9  (2.1-3.8) | 3.2  (2.3-4.2) | 3.5  (2.6-4.5) | 3.8  (2.8-4.9) | 4.1  (3.0-5.3) | 4.5  (3.3-5.9) | 5.0  (3.7-6.3) | 5.4  (4.0-6.9) | 5.9  (4.3-7.5) |
| 5 | 2.7  (2.0-3.6) | 3.1  (2.2-4.0) | 3.4  (2.5-4.4) | 3.9  (2.8-4.9) | 4.3  (3.1-5.4) | 4.7  (3.4-6.1) | 5.2  (3.8-6.8) | 5.8  (4.3-7.4) | 6.5  (4.8-8.3) | 7.2  (5.3-9.3) |
| Algorithm targeting all TB (CXR) | | | | | | | | | | |
| 1 | 14.8  (11.6-18.8) | 14.8  (11.2-18.7) | 14.8  (11.6-18.7) | 14.9  (11.6-19.0) | 15.0  (11.5-18.9) | 14.9  (11.4-18.8) | 15.0  (11.5-18.9) | 14.8  (11.5-19.1) | 15.1  (11.7-18.8) | 15.3  (11.6-18.9) |
| 2 | 15.7  (12.4-19.9) | 16.9  (12.9-21.3) | 18.2  (14.3-22.8) | 19.8  (15.4-25.0) | 21.7  (16.8-27.3) | 23.8  (18.4-29.7) | 26.5  (20.3-33.1) | 29.4  (23.3-37.3) | 34.2  (26.7-42.1) | 40.3  (30.9-49.6) |
| 3 | 16.8  (13.3-21.3) | 19.4  (15.0-24.4) | 22.5  (17.7-28.2) | 26.4  (20.6-33.3) | 31.6  (24.5-39.4) | 38.2  (29.5-47.5) | 47.1  (36.3-58.3) | 58.6  (45.8-73.9) | 76.7  (59.4-95.7) | 104.1  (77.6-133.7) |
| 4 | 18.0  (14.2-22.7) | 22.2  (17.2-27.9) | 27.8  (21.9-34.9) | 35.2  (27.7-44.5) | 46.0  (35.5-57.2) | 61.1  (46.8-75.7) | 83.0  (63.8-103.6) | 114.6  (88.6-147.6) | 169.7  (127.8-219.9) | 261.9  (190.8-361.5) |
| 5 | 19.1  (15.1-24.2) | 25.4  (19.7-31.9) | 34.0  (26.9-42.9) | 46.8  (36.6-59.0) | 66.5  (51.3-83.0) | 96.6  (73.4-121.3) | 144.9  (110.0-185.7) | 223.8  (168.3-296.9) | 374.1  (271.3-509.0) | 660.6  (460.2-974.2) |


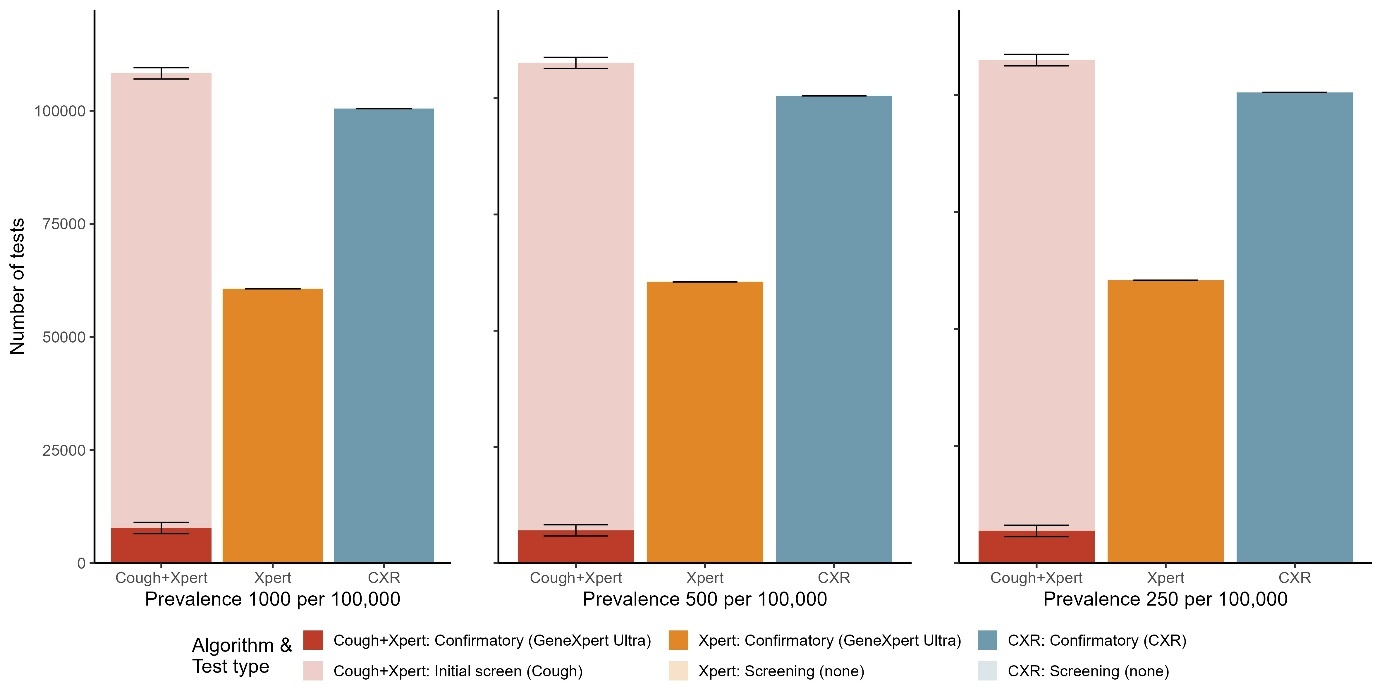


Fig C: Number of initial screen (light colour) and confirmatory (dark colour) tests required for one round of screening with 100% coverage by diagnostic algorithm in populations with different baseline prevalence levels.

Table L: Number of initial screen and confirmatory tests required for one round of screening with 100% coverage by diagnostic algorithm in populations with different baseline prevalence levels.


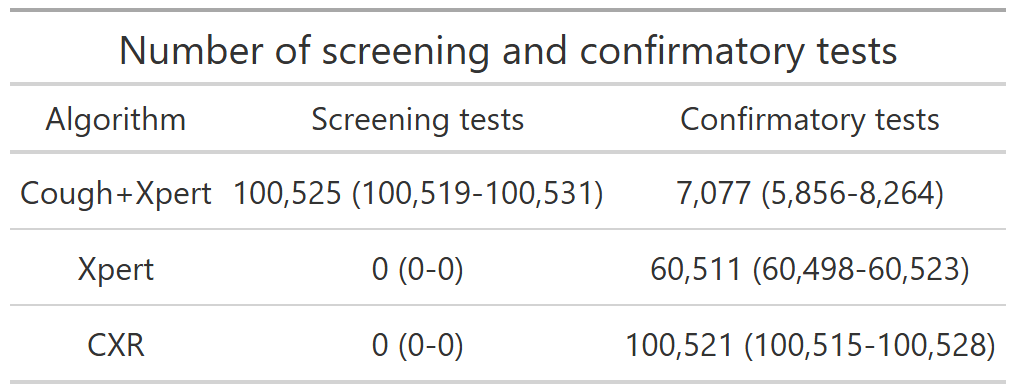


Table M: Projected percentage of symptomatic infectious TB episodes averted (2025-2035) by diagnostic algorithm, population coverage, and duration for a population with baseline prevalence of 500 per 100,000

|  | Projected reduction in symptomatic infectious TB episodes (2025-2035) | | | | | | | | | |
| --- | --- | --- | --- | --- | --- | --- | --- | --- | --- | --- |
|  | Population coverage | | | | | | | | | |
| Rounds | 10% | 20% | 30% | 40% | 50% | 60% | 70% | 80% | 90% | 100% |
| Algorithm targeting symptomatic infectious TB (Cough+Xpert) | | | | | | | | | | |
| 1 | 0.6% (0.5-0.7%) | 1.1% (0.9-1.4%) | 1.7% (1.4-2.0%) | 2.3% (1.9-2.7%) | 2.8% (2.3-3.4%) | 3.4% (2.8-4.1%) | 3.9% (3.2-4.8%) | 4.5% (3.7-5.5%) | 5.1% (4.2-6.1%) | 5.6% (4.7-6.8%) |
| 2 | 1.1% (0.9-1.3%) | 2.1% (1.7-2.5%) | 3.1% (2.6-3.7%) | 4.1% (3.4-5.0%) | 5.1% (4.2-6.1%) | 6.0% (5.0-7.2%) | 6.9% (5.8-8.3%) | 7.8% (6.6-9.3%) | 8.7% (7.3-10.5%) | 9.6% (7.9-11.5%) |
| 3 | 1.5% (1.2-1.8%) | 2.9% (2.4-3.5%) | 4.3% (3.6-5.2%) | 5.7% (4.7-6.9%) | 7.0% (5.8-8.3%) | 8.2% (6.9-9.8%) | 9.4% (7.9-11.2%) | 10.6% (8.9-12.8%) | 11.8% (9.9-13.9%) | 12.8% (10.8-15.3%) |
| 4 | 1.9% (1.5-2.3%) | 3.7% (3.1-4.4%) | 5.4% (4.5-6.5%) | 7.0% (5.8-8.4%) | 8.6% (7.2-10.3%) | 10.1% (8.5-12.0%) | 11.6% (9.7-13.8%) | 12.9% (10.9-15.4%) | 14.2% (12.1-16.9%) | 15.6% (13.0-18.4%) |
| 5 | 2.2% (1.8-2.7%) | 4.3% (3.5-5.2%) | 6.3% (5.2-7.5%) | 8.2% (6.8-9.7%) | 9.9% (8.3-11.8%) | 11.6% (9.9-13.9%) | 13.3% (11.3-15.7%) | 14.9% (12.4-17.8%) | 16.4% (13.9-19.3%) | 17.8% (15.1-20.9%) |
| Algorithm targeting infectious TB (Xpert) | | | | | | | | | | |
| 1 | 1.9% (1.7-2.3%) | 3.9% (3.3-4.5%) | 5.9% (5-6.8%) | 7.8% (6.6-8.9%) | 9.8% (8.4-11.3%) | 11.8% (10.1-13.6%) | 13.7% (11.9-15.9%) | 15.8% (13.4-18.2%) | 17.7% (15.1-20.6%) | 19.6% (16.9-22.8%) |
| 2 | 3.7% (3.1-4.2%) | 7.2% (6.3-8.3%) | 10.7% (9.2-12.4%) | 14% (12.2-16.2%) | 17.4% (15.1-19.9%) | 20.5% (17.8-23.4%) | 23.5% (20.4-26.9%) | 26.5% (22.9-30.0%) | 29.3% (25.5-33.3%) | 32% (28-36.1%) |
| 3 | 5.2% (4.5-6.0%) | 10.2% (8.7-11.7%) | 14.8% (12.8-17.0%) | 19.3% (16.7-21.9%) | 23.5% (20.5-26.7%) | 27.5% (23.9-30.9%) | 31.2% (27.5-34.9%) | 34.8% (30.4-39%) | 38.0% (33.2-42.6%) | 41.0% (36.3-45.8%) |
| 4 | 6.5% (5.6-7.5%) | 12.6% (11.0-14.4%) | 18.2% (15.8-20.9%) | 23.5% (20.6-26.7%) | 28.4% (24.8-32.1%) | 32.9% (29.0-37.1%) | 37.2% (33.1-41.4%) | 40.9% (36.0-45.5%) | 44.6% (39.9-49.2%) | 48.0% (42.9-52.5%) |
| 5 | 7.7% (6.6-8.8%) | 14.7% (12.8-16.7%) | 21.2% (18.3-24.1%) | 27.0% (23.6-30.3%) | 32.4% (28.5-36.3%) | 37.1% (33.1-41.2%) | 41.5% (36.9-46.4%) | 45.6% (40.9-50.2%) | 49.3% (44.3-53.9%) | 52.7% (47.9-57.5%) |
| Algorithm targeting all TB (CXR) | | | | | | | | | | |
| 1 | 5.0% (4.7-5.3%) | 10.1% (9.5-10.6%) | 15.2% (14.3-16.0%) | 20.3% (19.2-21.4%) | 25.4% (24.0-26.8%) | 30.6% (28.8-32.3%) | 35.9% (33.8-37.9%) | 41.1% (38.9-43.5%) | 46.5% (43.9-49.1%) | 51.8% (48.9-54.7%) |
| 2 | 9.3% (8.8-9.8%) | 18.1% (17.1-19.0%) | 26.5% (25.2-27.9%) | 34.3% (32.7-36.1%) | 41.8% (39.8-43.8%) | 48.6% (46.4-50.7%) | 55.0% (52.7-57.2%) | 60.8% (58.3-63.0%) | 66.1% (63.6-68.2%) | 70.7% (68.3-73.0%) |
| 3 | 12.9% (12.2-13.6%) | 24.5% (23.3-25.7%) | 34.9% (33.3-36.4%) | 44.0% (42.2-45.8%) | 52.1% (50.1-54.1%) | 59.1% (57.0-61.1%) | 65.0% (63.0-66.8%) | 69.9% (68.0-71.7%) | 74.0% (72.2-75.6%) | 77.2% (75.6-78.7%) |
| 4 | 15.9% (15.0-16.7%) | 29.4% (28.1-30.7%) | 40.9% (39.3-42.7%) | 50.6% (48.6-52.4%) | 58.3% (56.6-60.3%) | 64.8% (62.9-66.6%) | 69.9% (68.2-71.5%) | 73.9% (72.4-75.3%) | 77.1% (75.7-78.3%) | 79.5% (78.4-80.4%) |
| 5 | 18.2% (17.4-19.1%) | 33.2% (31.7-34.5%) | 45.1% (43.4-46.9%) | 54.8% (52.9-56.6%) | 62.2% (60.5-63.9%) | 67.9% (66.3-69.4%) | 72.4% (70.9-73.7%) | 75.7% (74.6-76.8%) | 78.2% (77.2-79.2%) | 80.2% (79.3-80.9%) |

Table N: Projected percentage of TB-associated deaths averted (2025-2035) by diagnostic algorithm, population coverage, and duration for a population with infectious TB prevalence of 500 per 100,000.

|  | Projected reduction in TB-associated deaths (2025-2035) | | | | | | | | | |
| --- | --- | --- | --- | --- | --- | --- | --- | --- | --- | --- |
|  | Population coverage | | | | | | | | | |
| Rounds | 10% | 20% | 30% | 40% | 50% | 60% | 70% | 80% | 90% | 100% |
| Algorithm targeting symptomatic infectious TB (Cough+Xpert) | | | | | | | | | | |
| 1 | 1.0% (0.9-1.2%) | 2.0% (1.8-2.3%) | 3.0% (2.6-3.5%) | 4.0% (3.5-4.6%) | 5.0% (4.4-5.8%) | 6.0% (5.3-7.0%) | 7.0% (6.1-8.1%) | 8.0% (7.0-9.2%) | 9.1% (7.8-10.5%) | 10.1% (8.7-11.6%) |
| 2 | 1.9% (1.7-2.2%) | 3.8% (3.3-4.3%) | 5.7% (4.9-6.5%) | 7.4% (6.5-8.6%) | 9.2% (8.1-10.5%) | 10.9% (9.6-12.5%) | 12.6% (11.0-14.3%) | 14.1% (12.6-16.1%) | 15.7% (13.9-18%) | 17.3% (15.2-19.8%) |
| 3 | 2.8% (2.4-3.2%) | 5.5% (4.8-6.3%) | 8.1% (7.1-9.2%) | 10.5% (9.3-12.1%) | 13.0% (11.4-14.8%) | 15.2% (13.5-17.3%) | 17.4% (15.5-19.6%) | 19.6% (17.3-22.3%) | 21.6% (19.3-24.2%) | 23.6% (21.1-26.4%) |
| 4 | 3.6% (3.2-4.1%) | 7.0% (6.2-8.0%) | 10.3% (9.1-11.7%) | 13.4% (11.8-15.3%) | 16.4% (14.5-18.4%) | 19.1% (17.0-21.5%) | 21.8% (19.4-24.6%) | 24.3% (21.9-27.5%) | 26.8% (23.9-30.0%) | 29.2% (26.1-32.5%) |
| 5 | 4.3% (3.8-4.9%) | 8.4% (7.4-9.7%) | 12.3% (10.8-13.9%) | 15.9% (14.2-18.0%) | 19.4% (17.2-21.8%) | 22.6% (20.5-25.4%) | 25.8% (23.0-28.7%) | 28.8% (25.6-32.2%) | 31.5% (28.3-34.9%) | 34.1% (31.0-37.7%) |
| Algorithm targeting infectious TB (Xpert) | | | | | | | | | | |
| 1 | 2.4% (2.1-2.7%) | 4.7% (4.1-5.3%) | 7.1% (6.2-8.0%) | 9.4% (8.2-10.5%) | 11.8% (10.5-13.2%) | 14.2% (12.5-15.9%) | 16.5% (14.6-18.6%) | 19% (16.6-21.3%) | 21.2% (18.8-24.1%) | 23.6% (21-26.8%) |
| 2 | 4.5% (3.9-5.0%) | 8.8% (7.8-9.8%) | 12.9% (11.5-14.6%) | 16.9% (15.1-19.0%) | 20.8% (18.6-23.2%) | 24.5% (22-27.4%) | 28% (25-31.3%) | 31.5% (28.1-34.8%) | 34.7% (31.2-38.5%) | 37.8% (34.1-41.6%) |
| 3 | 6.4% (5.7-7.1%) | 12.3% (11.0-13.9%) | 17.9% (16.0-20.0%) | 23.3% (20.7-25.7%) | 28.2% (25.4-31.1%) | 32.8% (29.7-35.9%) | 37.1% (33.9-40.6%) | 41.1% (37.3-44.8%) | 44.8% (40.8-48.8%) | 48.3% (44.1-52.5%) |
| 4 | 8.0% (7.1-9.0%) | 15.4% (13.9-17.2%) | 22.1% (20.0-24.7%) | 28.4% (25.8-31.4%) | 34.2% (30.9-37.4%) | 39.4% (35.9-43.2%) | 44.2% (40.7-47.9%) | 48.5% (44.3-52.5%) | 52.5% (48.6-56.5%) | 56.2% (52.1-60%) |
| 5 | 9.5% (8.5-10.6%) | 18.1% (16.3-20.1%) | 25.9% (23.3-28.6%) | 32.8% (29.8-35.9%) | 39.0% (35.8-42.5%) | 44.6% (41.1-48.3%) | 49.6% (45.7-53.7%) | 54.2% (50.2-58%) | 58.2% (54.2-62%) | 61.8% (58.1-65.5%) |
| Algorithm targeting all TB (CXR) | | | | | | | | | | |
| 1 | 5.2% (4.9-5.5%) | 10.4% (9.9-10.9%) | 15.7% (14.8-16.5%) | 21.0% (19.9-22.0%) | 26.2% (24.8-27.6%) | 31.5% (29.8-33.2%) | 37.0% (35.0-38.9%) | 42.3% (40.2-44.7%) | 47.8% (45.3-50.4%) | 53.2% (50.5-56.1%) |
| 2 | 9.6% (9.1-10.1%) | 18.7% (17.8-19.6%) | 27.3% (26.0-28.6%) | 35.3% (33.7-37.1%) | 43.0% (41.0-44.9%) | 49.9% (47.8-51.9%) | 56.4% (54.2-58.6%) | 62.3% (59.8-64.3%) | 67.5% (65.3-69.6%) | 72.2% (69.9-74.3%) |
| 3 | 13.3% (12.6-14%) | 25.3% (24.1-26.5%) | 35.9% (34.4-37.4%) | 45.3% (43.4-47.0%) | 53.4% (51.5-55.4%) | 60.6% (58.5-62.5%) | 66.5% (64.7-68.2%) | 71.4% (69.6-73.1%) | 75.5% (73.7-77.0%) | 78.7% (77.2-80.0%) |
| 4 | 16.4% (15.6-17.2%) | 30.4% (29.1-31.6%) | 42.1% (40.5-43.8%) | 52.0% (50.0-53.7%) | 59.9% (58.1-61.6%) | 66.3% (64.5-68.0%) | 71.5% (69.9-72.9%) | 75.5% (74.0-76.8%) | 78.6% (77.4-79.7%) | 81.0% (80.0-81.9%) |
| 5 | 18.9% (18.0-19.8%) | 34.3% (32.8-35.5%) | 46.5% (44.8-48.2%) | 56.2% (54.4-57.9%) | 63.7% (62.2-65.3%) | 69.5% (67.9-70.9%) | 73.9% (72.6-75.1%) | 77.3% (76.2-78.3%) | 79.7% (78.7-80.6%) | 81.7% (80.8-82.4%) |

Table O: Projected reduction in TB mortality in 2035 by diagnostic algorithm, population coverage, and duration for a population with infectious prevalence of 500 per 100,000.

|  | Projected reduction in 2035 TB mortality | | | | | | | | | |
| --- | --- | --- | --- | --- | --- | --- | --- | --- | --- | --- |
|  | Population coverage | | | | | | | | | |
| Rounds | 10% | 20% | 30% | 40% | 50% | 60% | 70% | 80% | 90% | 100% |
| Algorithm targeting symptomatic infectious TB (Cough+Xpert) | | | | | | | | | | |
| 1 | 0.7% (0.6-0.8%) | 1.4% (1.1-1.6%) | 2.1% (1.7-2.4%) | 2.8% (2.3-3.3%) | 3.4% (2.9-4.1%) | 4.1% (3.5-4.9%) | 4.8% (4.1-5.8%) | 5.5% (4.7-6.5%) | 6.2% (5.3-7.3%) | 6.9% (5.8-8.2%) |
| 2 | 1.4% (1.1-1.6%) | 2.7% (2.3-3.2%) | 4.0% (3.3-4.7%) | 5.3% (4.4-6.2%) | 6.6% (5.5-7.8%) | 7.8% (6.6-9.1%) | 8.9% (7.4-10.4%) | 10.1% (8.5-11.9%) | 11.2% (9.4-13.1%) | 12.3% (10.4-14.3%) |
| 3 | 2.1% (1.7-2.4%) | 4.0% (3.3-4.7%) | 6.0% (5.0-6.9%) | 7.7% (6.6-9.0%) | 9.5% (8.0-11.1%) | 11.2% (9.4-13.1%) | 12.8% (10.8-15.0%) | 14.4% (12.2-16.7%) | 15.9% (13.4-18.3%) | 17.3% (14.6-20.1%) |
| 4 | 2.7% (2.3-3.2%) | 5.3% (4.5-6.2%) | 7.8% (6.5-9.1%) | 10.2% (8.5-11.9%) | 12.4% (10.5-14.4%) | 14.5% (12.4-16.8%) | 16.6% (14-19.2%) | 18.5% (15.6-21.6%) | 20.4% (17.3-23.6%) | 22.1% (18.9-25.7%) |
| 5 | 3.4% (2.8-4.0%) | 6.6% (5.6-7.7%) | 9.7% (8.1-11.3%) | 12.6% (10.7-14.7%) | 15.3% (12.9-17.8%) | 17.9% (15.0-21.0%) | 20.2% (16.9-23.4%) | 22.4% (19.0-25.7%) | 24.7% (20.9-28.5%) | 26.7% (22.8-30.6%) |
| Algorithm targeting infectious TB (Xpert) | | | | | | | | | | |
| 1 | 1.8% (1.5-2.1%) | 3.7% (3.1-4.3%) | 5.5% (4.7-6.4%) | 7.4% (6.2-8.5%) | 9.3% (7.9-10.7%) | 11.2% (9.5-13.0%) | 13.0% (11.1-15.1%) | 15.0% (12.6-17.5%) | 16.9% (14.2-19.8%) | 18.8% (15.9-22.0%) |
| 2 | 3.6% (3.1-4.2%) | 7.2% (6.2-8.3%) | 10.7% (9.1-12.4%) | 14.0% (12.0-16.3%) | 17.4% (14.9-20.1%) | 20.5% (17.7-23.7%) | 23.6% (20.3-27.2%) | 26.7% (22.8-30.4%) | 29.5% (25.5-34.0%) | 32.3% (27.9-36.7%) |
| 3 | 5.5% (4.6-6.3%) | 10.7% (9.1-12.5%) | 15.6% (13.4-18.1%) | 20.5% (17.5-23.3%) | 24.9% (21.4-28.6%) | 29.1% (25.2-33.0%) | 33.2% (28.9-37.4%) | 36.9% (32.0-41.8%) | 40.5% (34.9-45.8%) | 43.7% (38.2-49.2%) |
| 4 | 7.3% (6.2-8.4%) | 14.1% (12.1-16.1%) | 20.4% (17.6-23.5%) | 26.3% (22.8-30.1%) | 31.8% (27.5-36.1%) | 36.9% (32.2-41.9%) | 41.6% (36.8-46.7%) | 45.8% (39.8-51.3%) | 49.9% (44.3-55.5%) | 53.7% (47.7-59.1%) |
| 5 | 9.1% (7.7-10.5%) | 17.4% (15.1-20.0%) | 25.0% (21.5-28.8%) | 32.0% (27.7-36.1%) | 38.2% (33.5-43.1%) | 43.9% (38.8-49.2%) | 49.0% (43.1-54.9%) | 53.7% (47.8-59.5%) | 57.9% (51.9-63.6%) | 61.8% (55.8-67.6%) |
| Algorithm targeting all TB (CXR) | | | | | | | | | | |
| 1 | 5.6% (5.3-5.9%) | 11.3% (10.6-11.9%) | 17% (16.1-18%) | 22.7% (21.5-24.1%) | 28.6% (27.0-30.3%) | 34.4% (32.6-36.5%) | 40.4% (38.2-42.8%) | 46.6% (43.9-49.2%) | 52.6% (49.5-55.6%) | 58.8% (55.6-62.2%) |
| 2 | 11.0% (10.3-11.6%) | 21.4% (20.3-22.6%) | 31.4% (29.8-33.0%) | 40.7% (38.8-42.8%) | 49.5% (47.1-51.8%) | 57.8% (55.1-60.2%) | 65.1% (62.5-67.8%) | 72.1% (69.1-74.7%) | 78.0% (75.4-80.9%) | 83.5% (80.6-86.1%) |
| 3 | 16.1% (15.3-17%) | 30.6% (29.2-32.1%) | 43.5% (41.5-45.5%) | 54.8% (52.5-57.2%) | 64.7% (62.1-67.0%) | 73.0% (70.5-75.4%) | 79.9% (77.5-82.2%) | 85.6% (83.4-87.9%) | 90.2% (88.1-91.9%) | 93.4% (91.8-95.0%) |
| 4 | 21.0%  (19.8-22.1%) | 38.9%  (37-40.7%) | 53.8%  (51.5-55.9%) | 65.9%  (63.4-68.2%) | 75.3%  (73.1-77.7%) | 83%  (80.8-84.9%) | 88.5%  (86.5-90.2%) | 92.6%  (91.1-94.0%) | 95.5%  (94.3-96.5%) | 97.4%  (96.5-98.2%) |
| 5 | 25.7% (24.4-27%) | 46.3% (44.2-48.3%) | 62.1% (60.0-64.3%) | 74.1% (72.0-76.4%) | 83% (80.9-84.9%) | 89.3% (87.5-90.8%) | 93.4% (92.1-94.7%) | 96.2% (95.2-97.1%) | 98.0% (97.3-98.5%) | 99.0% (98.5-99.3%) |


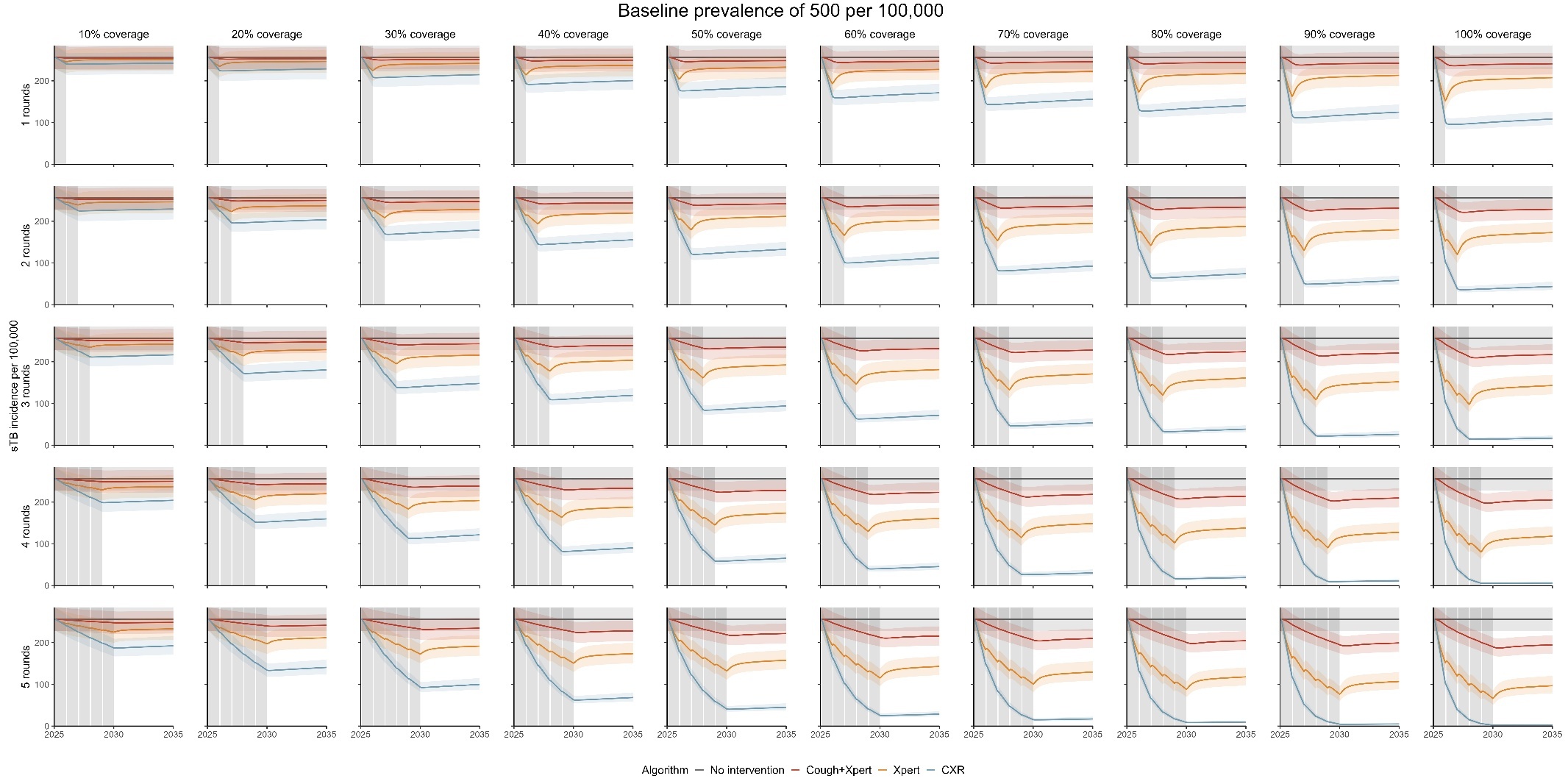


Fig D: Projected incidence of symptomatic TB by diagnostic algorithm, population coverage, and duration for a population with baseline prevalence of 500 per 100,000.


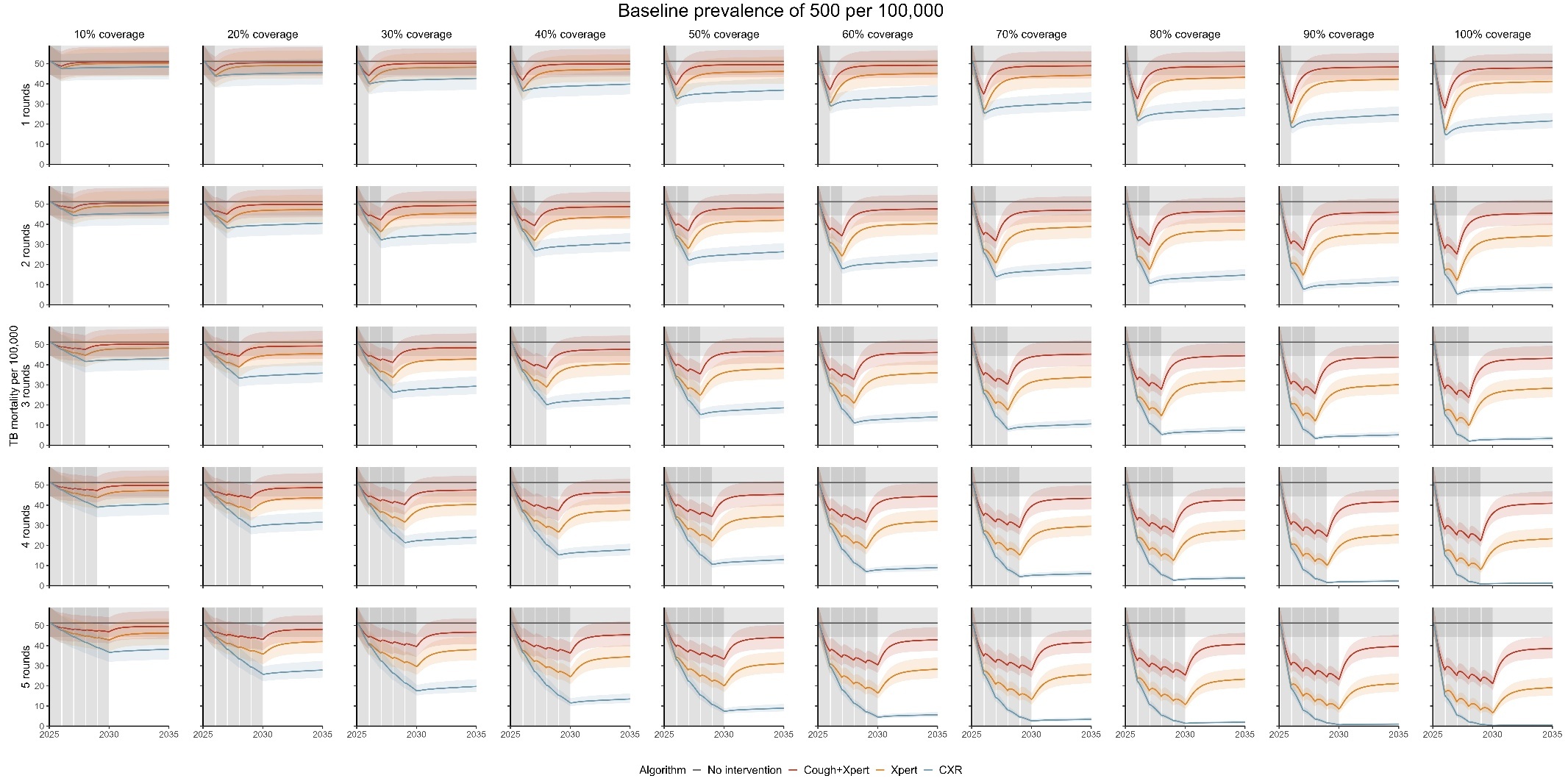


Fig E: Projected TB mortality by diagnostic algorithm, population coverage, and duration for a population with baseline prevalence of 500 per 100,000.


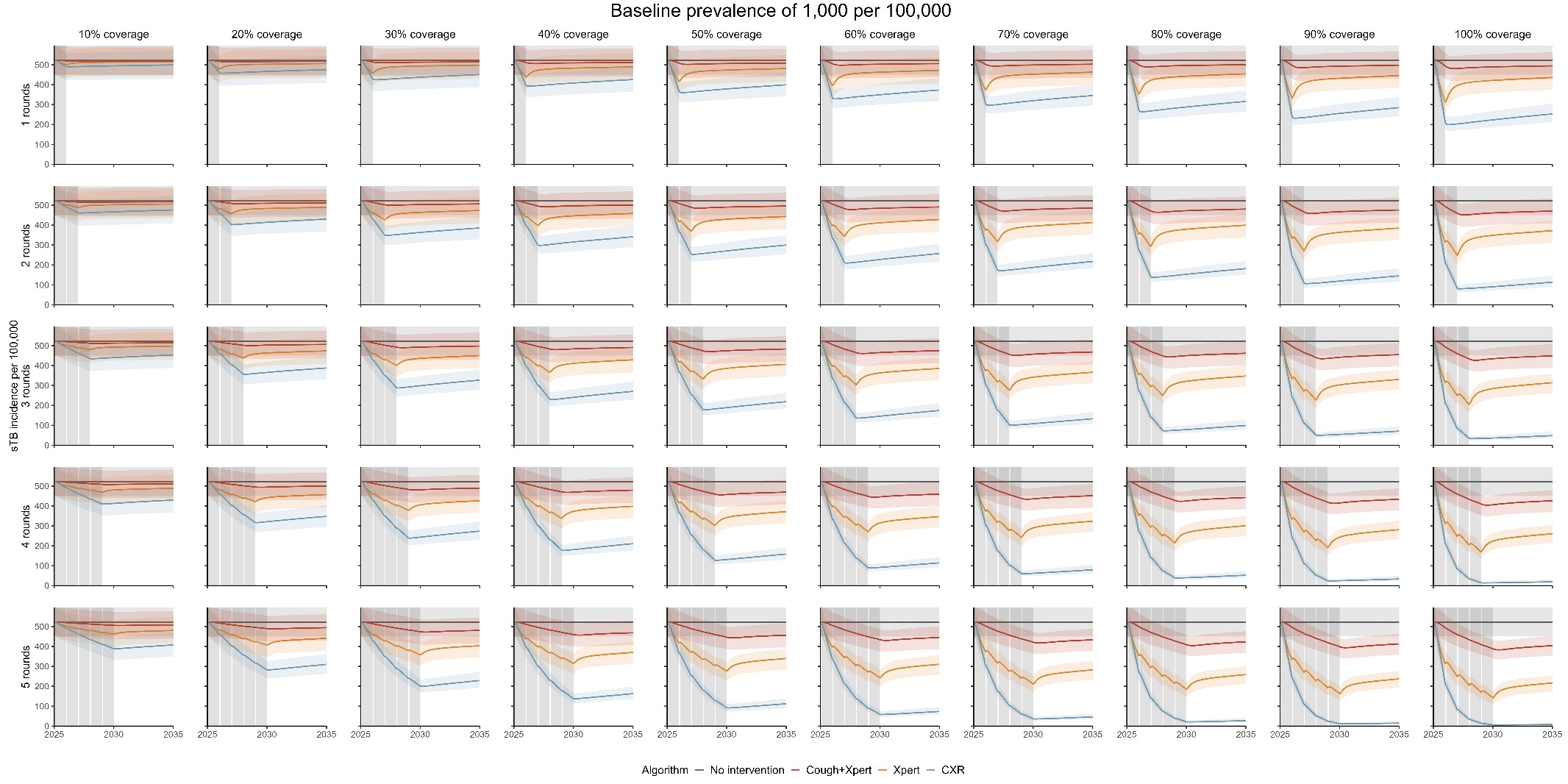


Fig F: Projected incidence of symptomatic TB by diagnostic algorithm, population coverage, and duration for a population with baseline prevalence of 1,000 per 100,000.


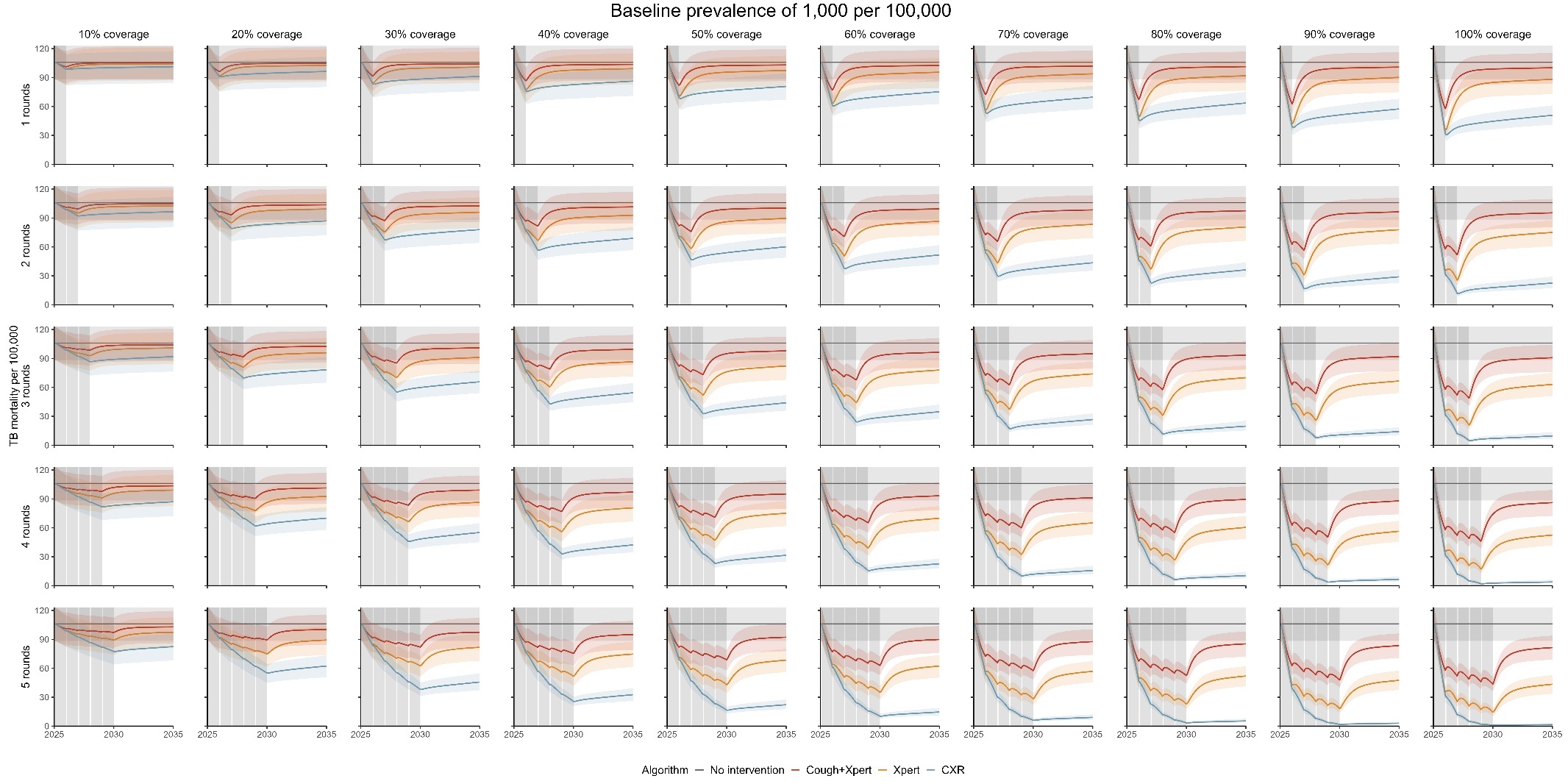


Fig G: Projected TB mortality by diagnostic algorithm, population coverage, and duration for a population with baseline prevalence of 1,000 per 100,000.


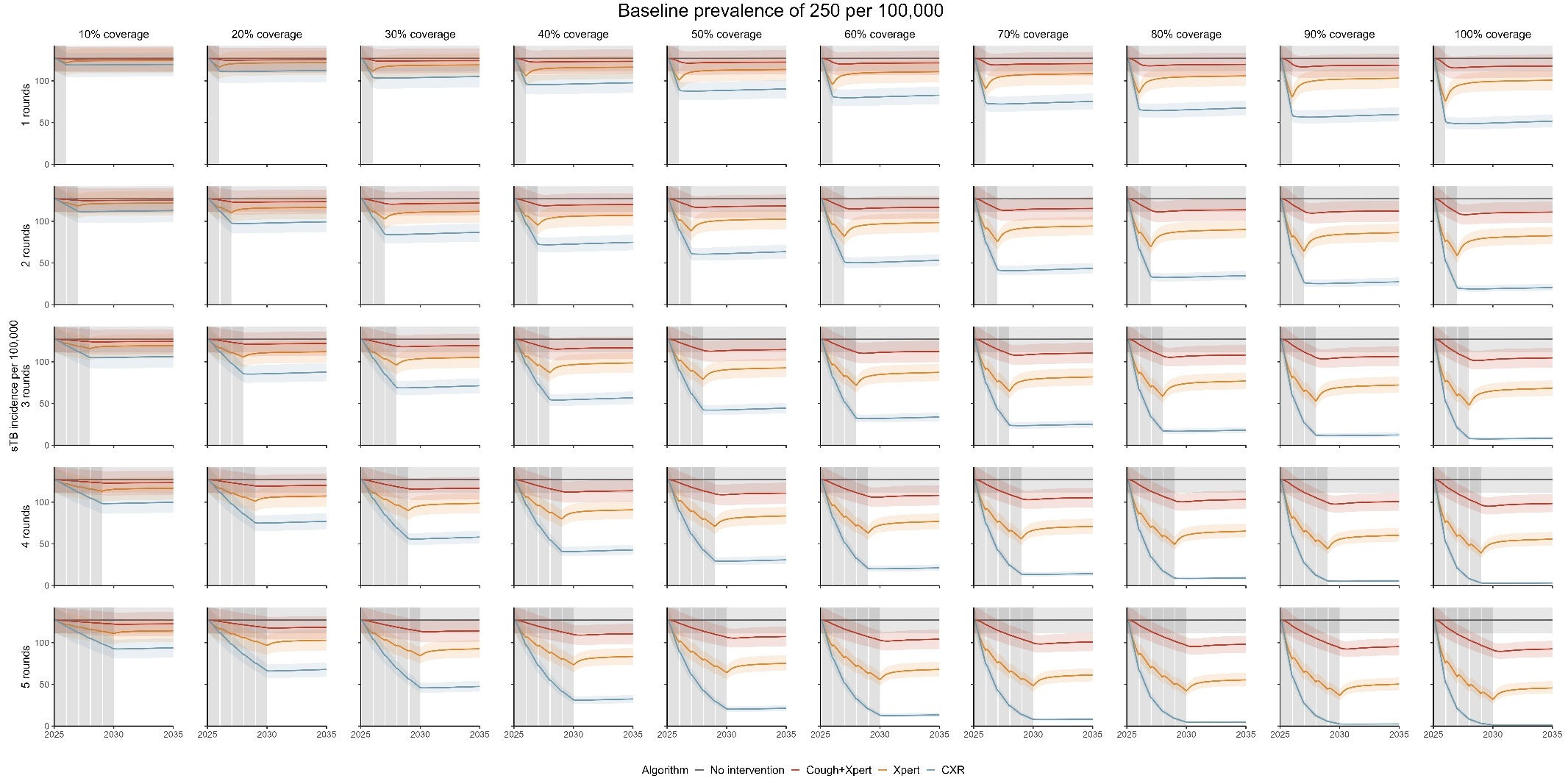


Fig H: Projected incidence of symptomatic TB by diagnostic algorithm, population coverage, and duration for a population with baseline prevalence of 250 per 100,000.


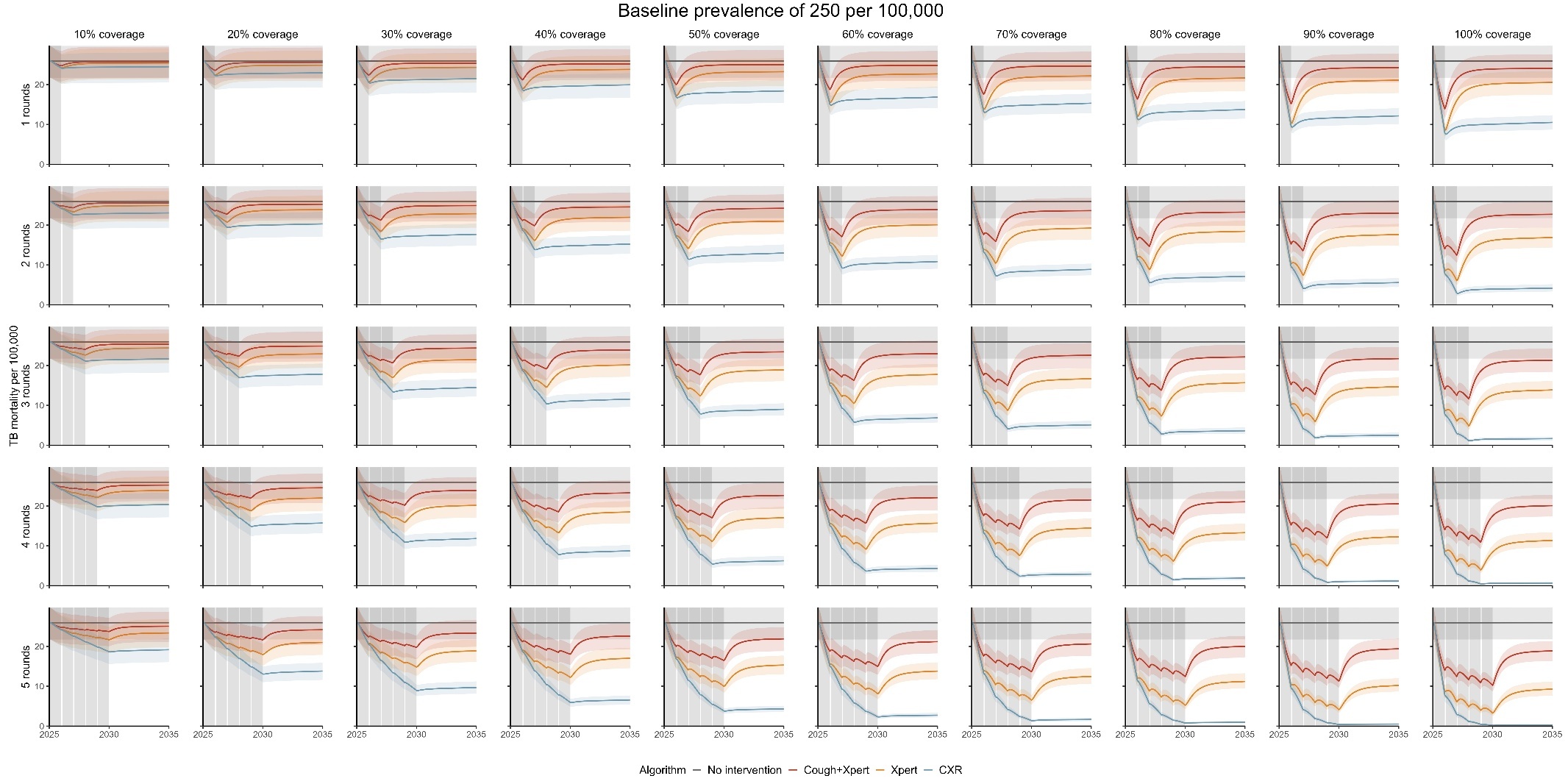


Fig I: Projected TB mortality by diagnostic algorithm, population coverage, and duration for a population with baseline prevalence of 250 per 100,000.

Table P: Projected reduction in symptomatic TB incidence at the end of screening by diagnostic algorithm, population coverage, and duration for baseline prevalence of 500 per 100,000 population.

|  | Projected reduction in symptomatic TB incidence at the end of screening | | | | | | | | | |
| --- | --- | --- | --- | --- | --- | --- | --- | --- | --- | --- |
|  | Population coverage | | | | | | | | | |
| Rounds | 10% | 20% | 30% | 40% | 50% | 60% | 70% | 80% | 90% | 100% |
| Algorithm targeting symptomatic infectious TB (Cough+Xpert) | | | | | | | | | | |
| 1 | 0.8% (0.7-1.0%) | 1.6% (1.3-1.9%) | 2.4% (2.0-2.9%) | 3.2% (2.6-3.9%) | 4.0% (3.2-4.8%) | 4.8% (3.9-5.8%) | 5.6% (4.6-6.7%) | 6.4% (5.2-7.7%) | 7.2% (5.9-8.7%) | 7.9% (6.5-9.7%) |
| 2 | 1.5% (1.2-1.8%) | 3.0% (2.5-3.6%) | 4.4% (3.7-5.4%) | 5.8% (4.8-7.1%) | 7.2% (6.0-8.7%) | 8.6% (7.1-10.3%) | 9.8% (8.1-11.8%) | 11.1% (9.2-13.3%) | 12.3% (10.2-14.9%) | 13.5% (11.1-16.2%) |
| 3 | 2.2% (1.8-2.6%) | 4.3% (3.5-5.1%) | 6.3% (5.2-7.5%) | 8.2% (6.8-10.0%) | 10.1% (8.3-12.0%) | 11.8% (9.9-14.1%) | 13.5% (11.3-16.0%) | 15.2% (12.7-18.2%) | 16.8% (14.1-19.9%) | 18.3% (15.4-21.8%) |
| 4 | 2.8% (2.3-3.4%) | 5.5% (4.6-6.6%) | 8.0% (6.7-9.7%) | 10.5% (8.7-12.5%) | 12.9% (10.7-15.3%) | 15.0% (12.6-17.7%) | 17.1% (14.3-20.3%) | 19.0% (15.9-22.5%) | 20.9% (17.6-24.7%) | 22.7% (19.0-26.8%) |
| 5 | 3.4% (2.8-4.2%) | 6.7% (5.4-8.1%) | 9.7% (8.0-11.6%) | 12.6% (10.5-15.0%) | 15.3% (12.8-18.2%) | 17.8% (15.2-21.2%) | 20.3% (17.2-23.9%) | 22.7% (18.9-26.9%) | 24.8% (21.0-29.2%) | 26.9% (22.8-31.5%) |
| Algorithm targeting infectious TB (Xpert) | | | | | | | | | | |
| 1 | 4.1% (3.6-4.6%) | 8.2% (7.2-9.2%) | 12.3% (10.7-13.8%) | 16.2% (14.3-18.4%) | 20.4% (17.8-22.9%) | 24.5% (21.5-27.4%) | 28.3% (25.2-32.0%) | 32.7% (28.5-36.6%) | 36.4% (32.2-41.2%) | 40.5% (35.8-45.5%) |
| 2 | 6.5% (5.6-7.4%) | 12.7% (11.1-14.4%) | 18.7% (16.4-21.1%) | 24.3% (21.5-27.5%) | 29.8% (26.3-33.6%) | 34.9% (30.9-39.3%) | 39.7% (35.2-44.8%) | 44.5% (39.5-49.5%) | 48.9% (43.5-54.3%) | 52.9% (47.6-58.7%) |
| 3 | 8.5% (7.4-9.7%) | 16.4% (14.3-18.8%) | 23.8% (20.8-26.9%) | 30.6% (26.8-34.4%) | 37.0% (32.7-41.3%) | 42.8% (37.8-47.5%) | 48.2% (43.2-53.2%) | 53.1% (47.4-58.6%) | 57.5% (51.5-63.1%) | 61.6% (55.6-67.4%) |
| 4 | 10.4% (9-11.9%) | 19.8% (17.3-22.4%) | 28.2% (24.9-32.1%) | 36.0% (32.0-40.5%) | 43.0% (38.1-48.2%) | 49.2% (44.0-54.6%) | 55.0% (49.8-60.3%) | 59.8% (53.9-65.5%) | 64.6% (58.8-69.8%) | 68.7% (62.7-73.9%) |
| 5 | 12.2% (10.6-13.9%) | 23.0% (20.2-26%) | 32.6% (28.5-36.9%) | 41.1% (36.3-45.6%) | 48.5% (43.2-53.7%) | 54.9% (49.6-60.1%) | 60.6% (54.7-66.4%) | 65.7% (59.9-71.1%) | 70.1% (64.2-75.4%) | 74.0% (68.5-79.1%) |
| Algorithm targeting all TB (CXR) | | | | | | | | | | |
| 1 | 6.3% (6.0-6.6%) | 12.7% (12.0-13.3%) | 18.9% (18.0-19.9%) | 25.2% (24-26.5%) | 31.4% (29.9-33.0%) | 37.7% (35.8-39.7%) | 44% (41.7-46.2%) | 50.1% (47.7-52.7%) | 56.4% (53.6-59.4%) | 62.6% (59.4-65.8%) |
| 2 | 12.2% (11.6-12.8%) | 23.5% (22.4-24.6%) | 34.1% (32.5-35.6%) | 43.8% (42.0-45.8%) | 52.9% (50.7-55.0%) | 60.9% (58.5-63.2%) | 68.3% (65.7-70.8%) | 75.0% (72.2-77.3%) | 80.7% (78.3-83.0%) | 85.7% (83.1-88.1%) |
| 3 | 17.5% (16.6-18.4%) | 32.9% (31.5-34.4%) | 46.2% (44.3-48.1%) | 57.6% (55.4-59.7%) | 67.3% (65.0-69.6%) | 75.5% (73.1-77.7%) | 82.0% (79.9-84.0%) | 87.2% (85.3-89.1%) | 91.4% (89.6-93.0%) | 94.4% (92.9-95.8%) |
| 4 | 22.5%  (21.4-23.6%) | 40.9%  (39.2-42.6%) | 55.9%  (53.9-58.1%) | 67.9%  (65.6-70.1%) | 77.2%  (75.1-79.3%) | 84.4%  (82.4-86.3%) | 89.7%  (88.0-91.2%) | 93.5%  (92.1-94.7%) | 96.1%  (95.0-97.1%) | 97.9%  (97.1-98.5%) |
| 5 | 27.0% (25.8-28.2%) | 48.0% (45.9-49.8%) | 63.8% (61.6-66.0%) | 75.7% (73.4-77.8%) | 84.1% (82.3-86%) | 90.1% (88.4-91.5%) | 94.1% (92.8-95.2%) | 96.7% (95.8-97.5%) | 98.3% (97.6-98.8%) | 99.2% (98.8-99.4%) |

Table Q: Projected reduction in TB mortality at the end of screening by diagnostic algorithm, population coverage, and duration for baseline prevalence of 500 per 100,000 population.

|  | Projected reduction in TB mortality at the end of screening | | | | | | | | | |
| --- | --- | --- | --- | --- | --- | --- | --- | --- | --- | --- |
|  | Population coverage | | | | | | | | | |
| Rounds | 10% | 20% | 30% | 40% | 50% | 60% | 70% | 80% | 90% | 100% |
| Algorithm targeting symptomatic infectious TB (Cough+Xpert) | | | | | | | | | | |
| 1 | 4.5% (4.1-4.9%) | 9.0% (8.2-9.9%) | 13.6% (12.4-14.9%) | 18.2% (16.5-19.8%) | 22.6% (20.8-24.8%) | 27.1% (24.7-29.6%) | 31.6% (28.7-34.5%) | 36.2% (33.0-39.5%) | 40.8% (36.8-44.6%) | 45.1% (41.0-49.4%) |
| 2 | 6.0% (5.4-6.7%) | 11.8% (10.7-13.1%) | 17.4% (15.8-19.3%) | 22.9% (20.8-25.3%) | 28.0% (25.5-30.6%) | 32.9% (30.1-36.2%) | 37.7% (34.5-41.2%) | 42.2% (38.8-46.1%) | 46.5% (42.6-50.5%) | 50.8% (46.7-55.0%) |
| 3 | 6.8% (6.1-7.7%) | 13.4% (12.0-14.8%) | 19.5% (17.6-21.5%) | 25.3% (22.8-28.1%) | 30.7% (27.9-33.8%) | 35.8% (32.6-39.2%) | 40.6% (37.1-44.2%) | 45.3% (41.4-49.2%) | 49.6% (45.7-53.7%) | 53.7% (49.8-57.7%) |
| 4 | 7.5% (6.7-8.5%) | 14.5% (13.0-16.3%) | 21.1% (19.0-23.5%) | 27.3% (24.6-30.1%) | 32.9% (30.0-36.3%) | 38.3% (34.7-41.7%) | 43.2% (39.6-47.2%) | 47.7% (44.0-52.0%) | 52.1% (48.0-56.5%) | 56.3% (51.9-60.7%) |
| 5 | 8.1% (7.2-9.2%) | 15.6% (13.9-17.6%) | 22.6% (20.2-25.2%) | 29.0% (26.2-32.1%) | 34.9% (31.6-38.5%) | 40.4% (37.1-44.4%) | 45.5% (41.6-49.6%) | 50.4% (45.8-54.9%) | 54.6% (50.4-59.1%) | 58.7% (54.5-63.2%) |
| Algorithm targeting infectious TB (Xpert) | | | | | | | | | | |
| 1 | 6.7% (6.3-7.1%) | 13.4% (12.6-14.2%) | 20.1% (19.0-21.3%) | 26.7% (25.3-28.2%) | 33.4% (31.5-35.3%) | 40.2% (37.9-42.5%) | 46.8% (44.3-49.4%) | 53.5% (50.4-56.6%) | 60.1% (56.8-63.4%) | 66.6% (63.1-70.4%) |
| 2 | 10.2% (9.5-11.0%) | 19.8% (18.5-21.2%) | 28.9% (27.0-30.9%) | 37.3% (35.0-39.9%) | 45.2% (42.4-47.8%) | 52.4% (49.5-55.6%) | 59.2% (55.7-62.8%) | 65.5% (62.1-68.9%) | 71.1% (67.6-74.6%) | 76.1% (72.6-79.7%) |
| 3 | 12.5% (11.5-13.5%) | 23.8% (21.9-25.9%) | 34.0% (31.5-36.7%) | 43.3% (40.2-46.2%) | 51.6% (48.2-54.9%) | 58.9% (55.4-62.4%) | 65.4% (62.0-69.1%) | 71.2% (67.3-74.7%) | 76.2% (72.4-79.6%) | 80.7% (77.3-84.0%) |
| 4 | 14.4% (13.1-15.6%) | 27.1% (25.0-29.4%) | 38.1% (35.3-41.4%) | 47.8% (44.5-51.4%) | 56.3% (52.4-60.1%) | 63.7% (59.9-67.7%) | 70.1% (66.3-74.0%) | 75.4% (71.4-79.1%) | 80.1% (76.8-83.5%) | 84.2% (80.7-87.1%) |
| 5 | 16.1% (14.7-17.7%) | 29.9% (27.4-32.7%) | 41.9% (38.4-45.3%) | 52.0% (48.1-55.7%) | 60.5% (56.7-64.6%) | 67.7% (63.8-71.6%) | 73.8% (69.8-77.8%) | 79.0% (75.2-82.5%) | 83.3% (79.7-86.4%) | 86.9% (83.9-89.7%) |
| Algorithm targeting all TB (CXR) | | | | | | | | | | |
| 1 | 7.2% (6.8-7.6%) | 14.4% (13.7-15.1%) | 21.5% (20.5-22.7%) | 28.7% (27.3-30.1%) | 35.9% (34.0-37.8%) | 43.1% (40.8-45.2%) | 50.0% (47.6-52.8%) | 57.3% (54.4-60.4%) | 64.2% (61.1-67.9%) | 71.4% (67.8-75.3%) |
| 2 | 13.3% (12.6-13.9%) | 25.5% (24.4-26.6%) | 36.8% (35.3-38.4%) | 47.1% (45.2-49.1%) | 56.6% (54.5-58.8%) | 65.0% (62.6-67.3%) | 72.6% (70.1-75.0%) | 79.2% (76.7-81.4%) | 84.8% (82.6-87.0%) | 89.5% (87.4-91.5%) |
| 3 | 18.6% (17.8-19.4%) | 34.8% (33.3-36.2%) | 48.6% (46.9-50.4%) | 60.4% (58.2-62.3%) | 70.2% (68.0-72.2%) | 78.2% (76.0-80.2%) | 84.6% (82.7-86.3%) | 89.5% (87.8-91.2%) | 93.3% (91.7-94.6%) | 96.0% (94.8-97.0%) |
| 4 | 23.6%  (22.5-24.7%) | 42.7%  (41.1-44.3%) | 58%  (56.1-60.1%) | 70.1%  (67.9-72.1%) | 79.2%  (77.3-81.1%) | 86.2%  (84.4-87.8%) | 91.2%  (89.7-92.5%) | 94.7%  (93.4-95.7%) | 97.0%  (96.1-97.8%) | 98.5%  (97.9-98.9%) |
| 5 | 28.1% (26.9-29.3%) | 49.7% (47.8-51.3%) | 65.6% (63.6-67.6%) | 77.3% (75.3-79.3%) | 85.6% (83.9-87.2%) | 91.2% (89.7-92.5%) | 95% (93.9-95.9%) | 97.3% (96.6-97.9%) | 98.7% (98.1-99.0%) | 99.4% (99.1-99.6%) |

Table R: Rebound in sTB incidence between the end of screening and 10 years after the start of screening by diagnostic algorithm, population coverage, and duration of screening for baseline prevalence of 500 per 100,000 population.

|  | Rebound in sTB incidence between the end of screening and 10 years after the start of screening | | | | | | | | | |
| --- | --- | --- | --- | --- | --- | --- | --- | --- | --- | --- |
|  | Population coverage | | | | | | | | | |
| Rounds | 10% | 20% | 30% | 40% | 50% | 60% | 70% | 80% | 90% | 100% |
| Symptomatic infectious TB (Cough+Xpert) | | | | | | | | | | |
| 1 | 25.3% (19.6-31.5%) | 25.1% (19.5-31.3%) | 25.1% (19.5-31.2%) | 25.0% (19.2-31.1%) | 24.9% (19.3-31.1%) | 24.9% (19.3-31.1%) | 24.7% (19.0-30.9%) | 24.7% (18.9-30.8%) | 24.5% (18.9-30.8%) | 24.5% (18.9-30.4%) |
| 2 | 21.5% (16.6-26.9%) | 21.2% (16.4-26.9%) | 21.0% (16.4-26.8%) | 20.9% (16.2-26.4%) | 20.7% (15.9-26.2%) | 20.5% (15.8-26.0%) | 20.4% (15.7-25.8%) | 20.2% (15.3-25.8%) | 20.0% (15.2-25.4%) | 19.8% (15.3-25.3%) |
| 3 | 17.7% (13.8-22.6%) | 17.5% (13.6-22.2%) | 17.2% (13.3-22.0%) | 17.1% (13.1-21.8%) | 16.8% (12.9-21.7%) | 16.6% (12.8-21.2%) | 16.4% (12.6-21.0%) | 16.2% (12.3-21.0%) | 16.0% (12.2-20.6%) | 15.7% (12.2-20.4%) |
| 4 | 14.6% (11.3-18.7%) | 14.3% (11.2-18.6%) | 14.0% (10.8-18.0%) | 13.8% (10.6-18.0%) | 13.6% (10.4-17.5%) | 13.4% (10.3-17.2%) | 13.1% (10.0-17.2%) | 12.9% (9.8-16.7%) | 12.7% (9.6-16.4%) | 12.5% (9.5-16.2%) |
| 5 | 11.9% (9.2-15.4%) | 11.6% (8.9-15.1%) | 11.3% (8.7-14.9%) | 11.1% (8.5-14.5%) | 10.9% (8.3-14.3%) | 10.6% (8.0-13.9%) | 10.4% (7.9-13.6%) | 10.2% (7.6-13.3%) | 10% (7.5-13.3%) | 9.8% (7.4-12.9%) |
| Infectious TB (Xpert) | | | | | | | | | | |
| 1 | 54.7% (50.7-58.3%) | 54.5% (50.7-58.4%) | 54.4% (50.3-58.0%) | 54% (49.8-57.9%) | 53.9% (49.8-57.9%) | 53.6% (49.4-57.6%) | 53.4% (49.3-57.7%) | 53.3% (49.2-57.2%) | 53% (49.1-57.2%) | 52.8% (48.6-57.0%) |
| 2 | 42.9% (39.5-46.2%) | 42.3% (38.9-45.8%) | 41.9% (38.4-45.5%) | 41.3% (38.1-44.8%) | 40.8% (37.5-44.5%) | 40.4% (37.0-43.9%) | 39.9% (36.4-43.6%) | 39.3% (35.9-43.0%) | 38.9% (35.5-42.7%) | 38.4% (35.1-41.9%) |
| 3 | 34.9% (31.8-38.1%) | 34.1% (31.0-37.1%) | 33.4% (30.1-36.5%) | 32.5% (29.7-35.5%) | 31.8% (28.9-35.0%) | 31% (27.9-34.2%) | 30.3% (27.4-33.7%) | 29.6% (26.6-33.0%) | 29.0% (26.0-32.5%) | 28.3% (25.0-31.6%) |
| 4 | 29.0% (26.3-31.7%) | 27.9% (25.3-30.9%) | 27.0% (24.4-29.9%) | 26.1% (23.4-28.8%) | 25.2% (22.5-28.1%) | 24.3% (21.6-27.2%) | 23.5% (20.6-26.4%) | 22.8% (20.0-25.7%) | 22.0% (19.3-24.9%) | 21.2% (18.5-24.0%) |
| 5 | 24.3% (22.1-26.8%) | 23.2% (20.8-25.8%) | 22.1% (19.7-24.6%) | 21.1% (18.8-23.7%) | 20.1% (17.8-22.8%) | 19.2% (16.9-21.8%) | 18.3% (16.0-21.0%) | 17.5% (15.1-20.2%) | 16.7% (14.4-19.3%) | 15.9% (13.5-18.6%) |
| All TB (CXR) | | | | | | | | | | |
| 1 | 15.5% (13.1-18.6%) | 14.8% (12.5-17.8%) | 14.0% (11.7-17.0%) | 13.2% (11-16.1%) | 12.5% (10.2-15.3%) | 11.5% (9.5-14.4%) | 10.7% (8.7-13.5%) | 9.8% (7.9-12.6%) | 8.9% (7.1-11.5%) | 7.9% (6.2-10.5%) |
| 2 | 13.4% (11.2-15.8%) | 12.1% (10.2-14.5%) | 10.9% (9.0-13.2%) | 9.6% (8.0-11.9%) | 8.4% (6.8-10.6%) | 7.4% (5.8-9.2%) | 6.3% (4.9-8.0%) | 5.2% (3.9-6.8%) | 4.1% (3.1-5.6%) | 3.2% (2.3-4.6%) |
| 3 | 11.2% (9.3-13.2%) | 9.7% (8.0-11.5%) | 8.1% (6.7-9.9%) | 6.8% (5.5-8.3%) | 5.5% (4.3-7.0%) | 4.3% (3.3-5.7%) | 3.4% (2.5-4.5%) | 2.5% (1.8-3.4%) | 1.7% (1.2-2.5%) | 1.1% (0.7-1.8%) |
| 4 | 9.1% (7.6-10.8%) | 7.4% (6.1-8.9%) | 5.9% (4.7-7.2%) | 4.5% (3.6-5.7%) | 3.4% (2.6-4.4%) | 2.5% (1.8-3.3%) | 1.7% (1.2-2.4%) | 1.1% (0.7-1.6%) | 0.6% (0.4-1.0%) | 0.4% (0.2-0.6%) |
| 5 | 7.1% (5.8-8.6%) | 5.4% (4.4-6.6%) | 4% (3.2-5.0%) | 2.9% (2.2-3.7%) | 2.0% (1.4-2.7%) | 1.3% (0.9-1.8%) | 0.8% (0.5-1.2%) | 0.4% (0.3-0.7%) | 0.2% (0.1-0.4%) | 0.1% (0.1-0.2%) |

Table S: Rebound in TB mortality between the end of screening and 10 years after the start of screening by diagnostic algorithm, population coverage, and duration of screening for baseline prevalence of 500 per 100,000 population.

|  | Rebound in TB mortality between the end of screening and 10 years after the start of screening | | | | | | | | | |
| --- | --- | --- | --- | --- | --- | --- | --- | --- | --- | --- |
|  | Population coverage | | | | | | | | | |
| Rounds | 10% | 20% | 30% | 40% | 50% | 60% | 70% | 80% | 90% | 100% |
| Symptomatic infectious TB (Cough+Xpert) | | | | | | | | | | |
| 1 | 86.6% (84.4-88.6%) | 86.7% (84.5-88.6%) | 86.7% (84.4-88.5%) | 86.6% (84.4-88.6%) | 86.6% (84.3-88.5%) | 86.6% (84.4-88.5%) | 86.5% (84.3-88.5%) | 86.5% (84.2-88.5%) | 86.5% (84.2-88.4%) | 86.5% (84.2-88.5%) |
| 2 | 80.1% (77.2-82.6%) | 79.9% (76.9-82.5%) | 79.7% (76.7-82.4%) | 79.5% (76.5-82.4%) | 79.4% (76.5-82%) | 79.2% (76.1-81.9%) | 79.0% (76.1-81.8%) | 78.9% (75.7-81.6%) | 78.7% (75.4-81.5%) | 78.4% (75.2-81.1%) |
| 3 | 73.6% (70.3-76.6%) | 73.4% (69.9-76.5%) | 73.0% (69.6-76.1%) | 72.8% (69.3-76.0%) | 72.5% (69.0-75.6%) | 72.2% (68.6-75.7%) | 71.8% (68.3-75.1%) | 71.5% (67.8-74.9%) | 71.3% (67.6-74.8%) | 71.0% (67.1-74.6%) |
| 4 | 67.8% (64.0-71.2%) | 67.4% (63.7-70.8%) | 66.9% (63.0-70.5%) | 66.5% (62.7-70.2%) | 66.1% (62.2-69.8%) | 65.8% (61.8-69.6%) | 65.4% (61.3-69.4%) | 64.9% (60.8-69.0%) | 64.7% (60.4-68.7%) | 64.3% (60.2-68.5%) |
| 5 | 62.4% (58.5-66.1%) | 61.8% (57.7-65.8%) | 61.5% (57.4-65.4%) | 60.9% (56.9-64.9%) | 60.5% (56.4-64.6%) | 60% (55.5-64.0%) | 59.7% (55.1-63.7%) | 59.1% (54.3-63.6%) | 58.6% (54.0-63.0%) | 58.2% (53.6-63.0%) |
| Infectious TB (Xpert) | | | | | | | | | | |
| 1 | 71.9% (68.1-75.5%) | 71.9% (68.4-75.5%) | 71.8% (67.9-75.4%) | 71.6% (68.2-75.4%) | 71.6% (68.0-75.2%) | 71.5% (67.8-75.1%) | 71.4% (67.8-75.0%) | 71.3% (67.6-75.1%) | 71.2% (67.4-75.1%) | 71.0% (67.4-74.8%) |
| 2 | 63.2% (59.5-67.0%) | 62.7% (58.7-66.5%) | 62.0% (58.1-66.0%) | 61.4% (57.5-65.4%) | 60.6% (56.6-65.1%) | 60.0% (56.0-64.2%) | 59.2% (55.1-63.4%) | 58.4% (54.3-62.9%) | 57.6% (53.3-61.8%) | 56.7% (52.3-61.3%) |
| 3 | 55.0% (51.3-59.1%) | 54.0% (50.0-58.1%) | 52.8% (49.0-57.4%) | 51.7% (47.9-56.1%) | 50.6% (46.3-55.0%) | 49.4% (45.5-54.0%) | 48.4% (44.4-52.8%) | 47.1% (42.8-51.9%) | 45.9% (41.5-51.0%) | 44.8% (40.1-49.9%) |
| 4 | 48% (44.4-51.7%) | 46.6% (42.7-50.8%) | 45.2% (41.2-49.5%) | 43.8% (39.7-48.0%) | 42.3% (38.3-46.8%) | 40.8% (36.6-45.5%) | 39.5% (35.4-44.0%) | 38.2% (33.6-43.2%) | 36.8% (32.4-41.6%) | 35.3% (30.9-40.2%) |
| 5 | 41.9% (38.4-45.9%) | 40.3% (36.9-44.3%) | 38.7% (34.6-43.3%) | 37.1% (33.3-41.5%) | 35.4% (31.5-39.9%) | 33.9% (29.8-38.3%) | 32.4% (28.2-37.2%) | 30.9% (26.6-35.7%) | 29.4% (25.1-34.2%) | 27.9% (23.6-32.8%) |
| All TB (CXR) | | | | | | | | | | |
| 1 | 24.7% (19.9-29.8%) | 24.1% (19.3-29.1%) | 23.4% (18.4-28.5%) | 22.7% (17.9-28.0%) | 22.5% (17.4-27.4%) | 21.8% (16.7-26.6%) | 20.8% (15.6-26.2%) | 20.3% (15.2-25.3%) | 19.4% (14.4-24.5%) | 18.8% (13.7-23.7%) |
| 2 | 19.6% (15.7-23.2%) | 18.1% (14.3-21.7%) | 16.7% (13.3-20.2%) | 15.3% (12.0-18.7%) | 13.9% (10.7-17.4%) | 12.6% (9.5-15.8%) | 11.2% (8.5-14.3%) | 9.8% (6.9-12.6%) | 8.3% (5.9-11.1%) | 7.0% (4.7-9.7%) |
| 3 | 15.5% (12.4-18.6%) | 13.9% (11.2-16.4%) | 12.0% (9.5-14.6%) | 10.4% (8.1-12.8%) | 8.8% (6.7-11.2%) | 7.3% (5.5-9.4%) | 6.0% (4.3-7.7%) | 4.6% (3.2-6.2%) | 3.5% (2.3-5.0%) | 2.6% (1.6-3.7%) |
| 4 | 12.5% (10.3-14.9%) | 10.5% (8.5-12.6%) | 8.6% (6.7-10.6%) | 6.9% (5.3-8.8%) | 5.5% (4.0-7.1%) | 4.2% (2.9-5.5%) | 3.1% (2.1-4.2%) | 2.2% (1.4-3.0%) | 1.4% (0.9-2.2%) | 0.9% (0.5-1.4%) |
| 5 | 9.9% (8.1-12.0%) | 7.8% (6.2-9.6%) | 6.1% (4.7-7.7%) | 4.6% (3.4-6.0%) | 3.3% (2.4-4.5%) | 2.3% (1.6-3.2%) | 1.6% (1.0-2.2%) | 1.0% (0.6-1.5%) | 0.6% (0.3-0.9%) | 0.3% (0.2-0.5%) |

**
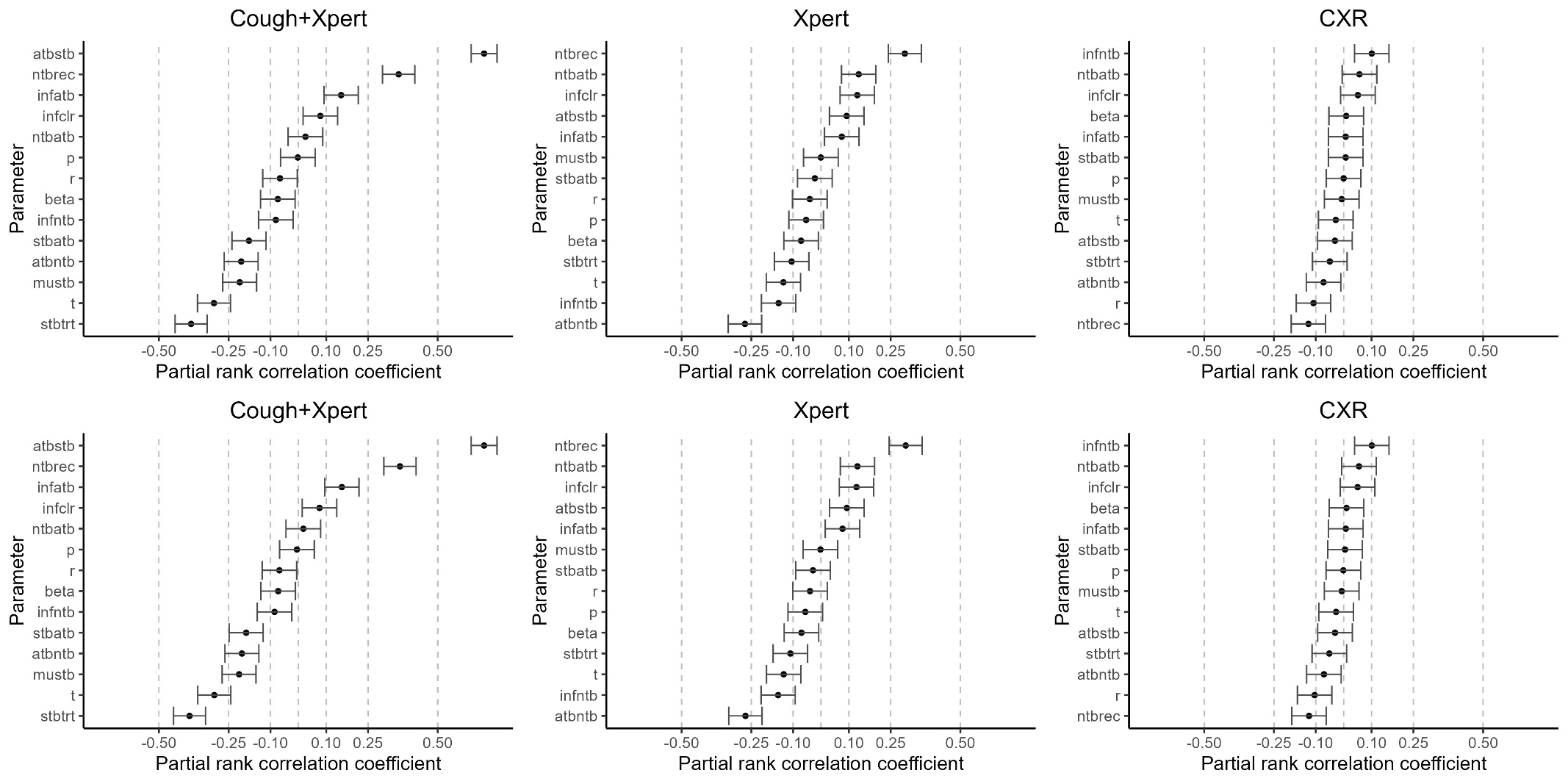
**

Fig J: Partial rank correlation coefficients for baseline prevalence of 500 per 100,000 population.

**
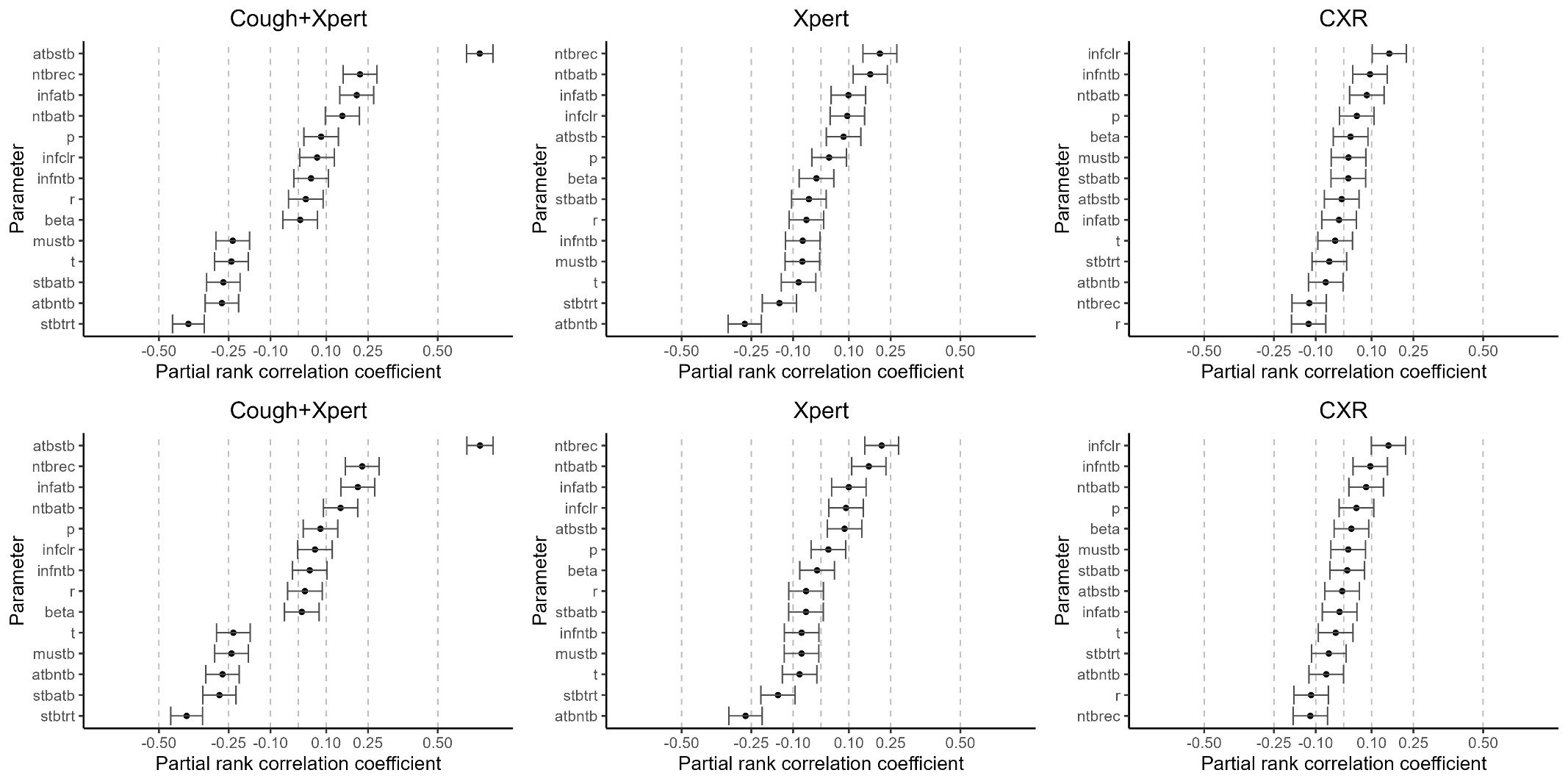
**

Fig K: Partial rank correlation coefficients for baseline prevalence of 1,000 per 100,000 population.

**
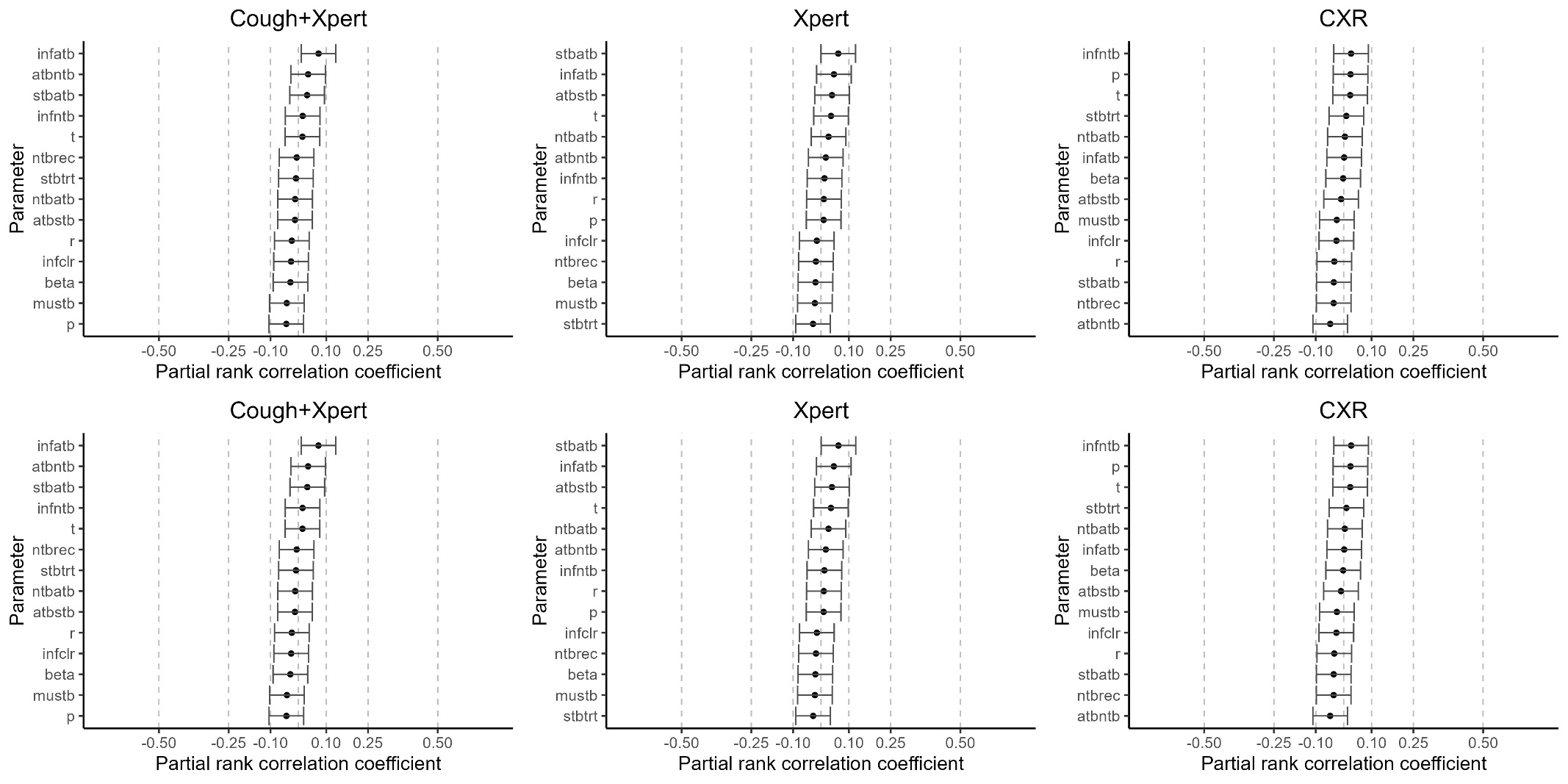
**

Fig L: Partial rank correlation coefficients for baseline prevalence of 250 per 100,000 population.

**References**

1. Andrews JR, Noubary F, Walensky RP, Cerda R, Losina E, Horsburgh CR. Risk of progression to active tuberculosis following reinfection with Mycobacterium tuberculosis. Clinical Infectious Diseases. 2012;54(6):784-91.

2. Verver S, Warren RM, Beyers N, Richardson M, Van Der Spuy GD, Borgdorff MW, et al. Rate of reinfection tuberculosis after successful treatment is higher than rate of new tuberculosis. American Journal of Respiratory and Critical Care Medicine. 2005;171(12):1430-5.

3. Emery JC, Dodd PJ, Banu S, Frascella B, Garden FL, Horton KC, et al. Estimating the contribution of subclinical tuberculosis disease to transmission: An individual patient data analysis from prevalence surveys. Elife. 2023;12:e82469.

4. Horton KC, Richards A, Emery JC, Esmail H, Houben RM. Reevaluating progression and pathways following Mycobacterium tuberculosis infection within the spectrum of tuberculosis. Proceedings of the National Academy of Sciences. 2023;120(47):e2221186120.

5. World Health Organization. Global tuberculosis report 2022. Geneva, Switzerland: World Health Organization, 2022 19 November 2022. Report No.

6. World Health Organization. WHO consolidated guidelines on tuberculosis. Module 4: treatment-drug-susceptible tuberculosis treatment: World Health Organization; 2022.

7. United Nations Department of Economic and Social Affairs. World Population Prospects 2022 2023. Available from: <https://population.un.org/wpp/>.

8. Soetaert K, Petzoldt T, Setzer RW. Solving differential equations in R: package deSolve. Journal of statistical software. 2010;33:1-25.

9. World Health Organization. WHO consolidated guidelines on tuberculosis. Module 2: screening-systematic screening for tuberculosis disease: World Health Organization; 2021.

10. World Health Organization. National tuberculosis prevalence surveys: what diagnostic algorithms should be used in future? Geneva, Switzerland: 2023.

11. Zifodya JS, Kreniske JS, Schiller I, Kohli M, Dendukuri N, Schumacher SG, et al. Xpert Ultra versus Xpert MTB/RIF for pulmonary tuberculosis and rifampicin resistance in adults with presumptive pulmonary tuberculosis. Cochrane Database of Systematic Reviews. 2021;(2).

12. Kendall EA, Kitonsa PJ, Nalutaaya A, Erisa KC, Mukiibi J, Nakasolya O, et al. The spectrum of tuberculosis disease in an urban Ugandan community and its health facilities. Clinical Infectious Diseases. 2021;72(12):e1035-e43.

13. Frascella B, Richards AS, Sossen B, Emery JC, Odone A, Law I, et al. Subclinical Tuberculosis Disease—A Review and Analysis of Prevalence Surveys to Inform Definitions, Burden, Associations, and Screening Methodology. Clinical Infectious Diseases. 2021;73(3):e830-e41.

14. Mungai B, Ong ‘angò J, Ku CC, Henrion MY, Morton B, Joekes E, et al. Accuracy of computer-aided chest X-ray in community-based tuberculosis screening: Lessons from the 2016 Kenya National Tuberculosis Prevalence Survey. PLOS Global Public Health. 2022;2(11):e0001272.
